# In-Frame and Frame-Shift Editing of the *Ehd1* Gene to Develop *Japonica* Rice With Prolonged Basic Vegetative Growth Periods

**DOI:** 10.3389/fpls.2020.00307

**Published:** 2020-03-19

**Authors:** Mingji Wu, Huaqing Liu, Yan Lin, Jianmin Chen, Yanping Fu, Jiami Luo, Zhujian Zhang, Kangjing Liang, Songbiao Chen, Feng Wang

**Affiliations:** ^1^College of Agriculture, Fujian Agricultural and Forestry University, Fuzhou, China; ^2^Fujian Key Laboratory of Genetic Engineering for Agriculture, Biotechnology Research Institute, Fujian Academy of Agricultural Sciences, Fuzhou, China; ^3^Marine and Agricultural Biotechnology Laboratory, Institute of Oceanography, Minjiang University, Fuzhou, China

**Keywords:** *japonica* rice, basic vegetative growth, heading date, CRISPR/Cas9, *Ehd1*, in-frame, frame-shift

## Abstract

*Japonica* rice has become increasingly popular in China owing to its superior grain quality. Over the past decades, “*indica* to *japonica*” projects have been proposed to promote cultivation of *japonica* rice in low latitudes in China. Traditionally, *japonica* varieties were planted mainly in mid latitudes in the northeast plain and Yangtze River region. The key obstacle for introducing elite mid-latitude *japonica* varieties to low latitudes is the severe shortening of growth period of the *japonica* varieties due to their sensitivity to low-latitude short photoperiod and high temperature. Here we report development of new *japonica* rice with prolonged basic vegetative growth (BVG) periods for low latitudes by targeted editing the *Early heading date 1* (*Ehd1*) gene. Using CRISPR/Cas9 system, we generated both frame-shift and/or in-frame deletion mutants in four *japonica* varieties, Nipponbare, Longdao16, Longdao24, and Xiushui134. When planting at low-latitude stations, the frame-shift homozygous lines exhibited significantly longer BVG periods compared with wild-types. Interestingly, we observed that minor deletion of the first few residues within the receiver domain could quantitatively impair the function of Ehd1 on activation of *Hd3a* and *RFT1*, resulting in an intermediate-long BVG period phenotype in the homozygous in-frame deletion *ehd1* lines. Field investigation further showed that, both the in-frame and frame-shift lines exhibited significantly improved yield potential compared with wild-types. Our study demonstrates an effective approach to rapid breeding of elite *japonica* varieties with intermediate-long and long BVG periods for flexible cropping systems in diverse areas or under different seasons in southern China, and other low-latitude regions.

## Introduction

Rice (*Oryza sativa* L.) is one of the most important crops in China. Cultivated rice is comprised of *japonica* and *indica* subspecies. Compared to *indica* varieties, *japonica* varieties in general have more superior grain quality – higher head rice rates and gel consistency, lower chalky rice rates, chalkiness degree, and amylose contents (Feng et al., 2017). Over the past decades, *japonica* rice has become increasingly popular in China. To meet the demands of consumers, “*indica* to *japonica*” projects have been proposed to promote cultivation of *japonica* varieties in southern China (Zhang et al., 2013; Leng et al., 2019).

Traditionally, *japonica* varieties were planted mainly in mid latitudes in the northeast plain and Yangtze River region, and *indica* varieties were planted in low latitudes in southern China (Min et al., 2011; Wang et al., 2015). Over the past decades, many elite *japonica* varieties have been bred for mid latitudes in China. However, most of the mid-latitude *japonica* varieties were sensitive to photoperiod or temperature, and they were not suitable for cultivating in low-latitude regions. Under short photoperiod and high temperature environmental conditions in low latitudes, the mid-latitude *japonica* varieties commonly display early flowering, resulting in low grain yield (Saito et al., 2009; Wei et al., 2009; Yuan et al., 2009).

The rice *Early heading date 1* (*Ehd1*) gene encodes a highly conserved B-type two component response regulator (RR), which acts as a key signal integrator in the networks regulating floral transition (Doi et al., 2004). Ehd1 promotes the expression of *Heading date 3a* (*Hd3a*) and *Rice Flowering Locust1* (*RFT1*) that induce rice flowering under both short day (SD) and long day (LD) conditions (Doi et al., 2004; Komiya et al., 2008). The expression of *Ehd1* is activated or repressed by multiple genes (Komiya et al., 2009; Shrestha et al., 2014). For example, *LVP1*/*SDG724* (Sun et al., 2012), *Ehd2*/*RID1*/*OsID1* (Matsubara et al., 2008; Park et al., 2008; Wu et al., 2008; Hu et al., 2013), *Ehd3* (Matsubara et al., 2011), *Ehd4* (Gao et al., 2013), *OsMADS50*/*DTH3* (Lee et al., 2004; Bian et al., 2011), OsMADS51 (Kim et al., 2007), *Hd-q*/*Ef7* (Yuan et al., 2009; Zhao et al., 2013) can positively regulate *Ehd1* transcription. In contrast, *Ehd1* expression is negatively regulated by *HD1* (Yano et al., 2000; Nemoto et al., 2016), *Ghd7* (Xue et al., 2008), *DTH7/Ghd7.1/OsPRR37* (Koo et al., 2013; Yan et al., 2013; Gao et al., 2014), *DTH8*/*EF8*/*Ghd8* (Wei et al., 2010; Yan et al., 2011; Feng et al., 2014) under LD, and negatively regulated by *SE5* under both LD and SD (Andrés et al., 2009). Mutation in *Ehd1* has a severe effect on the floral transition. A Gly-to-Arg point mutation in the GARP motif of Ehd1 leads to a reduced response to photoperiod in rice (Doi et al., 2004). This *ehd1* mutation allele enabled *japonica* varieties from Taiwan region suitable for planting under low-latitude conditions. These *japonica* varieties exhibit long basic vegetative growth (BVG) periods and display elite agronomic traits in Taiwan region (Nishida et al., 2002; Saito et al., 2009; Wei et al., 2016). Overall, these previous studies demonstrated the potentiality of *Ehd1* as a target for genome editing for regulating heading date in rice.

CRISPR/Cas9 technology has emerged as a powerful approach for rice research and breeding (Shan et al., 2014; Xie et al., 2015; Lu et al., 2017; Xu et al., 2017; Li et al., 2018). CRISPR/Cas9-mediated gene modification has been applied in rice for improvement of important agronomic traits, such as photo-sensitive and thermo-sensitive genic male sterility (Li et al., 2016; Zhou et al., 2016), herbicide-resistance (Sun et al., 2016), resistance to blast and tungro spherical virus (Wang et al., 2016; Macovei et al., 2018), low cadmium accumulation (Tang et al., 2017), grain yield (Miao et al., 2018), glutinous trait (Zhang J. et al., 2018), chilling tolerance (Mao et al., 2019), red pericarp (Zhu et al., 2019), and fixation of heterosis (Khanday et al., 2019; Wang et al., 2019). More recently, Cui et al. (2019) assessed the effect of ten heading time genes on reproductive transition and yield components in rice using a CRISPR/Cas9 system, revealing that heading time was often associated with yield-related traits. In the present study, we report development of new *japonica* rice exhibiting prolonged BVG periods for low latitudes by targeted editing the receiver domain region of Ehd1. Using CRISPR/Cas9 system, we generated both frame-shift and/or in-frame deletion mutants in four *japonica* varieties, Nipponbare, Longdao16, Longdao24, and Xiushui134. When planting under low-latitude conditions, the frame-shift mutants of Nipponbare, Longdao16, Longdao24, and Xiushui134 exhibited significantly longer BVG periods compared with their wild-type parents. Interestingly, the in-frame mutants of Longdao16, Longdao24, and Xiushui134 possessed intermediate-long BVG periods. Field investigation further showed that, both the in-frame and frame-shift mutant lines showed significantly improved yield potential compared with wild-type plants. Our study demonstrates an effective approach for breeding new *japonica* varieties with intermediate-long and more extremely long BVG periods for flexible cropping systems in southern China, as well as in other low-latitude regions.

## Materials and Methods

### Plant Materials

The *japonica* variety Nipponbare and three elite *japonica* varieties Longdao16, Longdao24 and Xiushui134 were used in this study.

### Construction of sgRNA-Cas9 Plant Expression Vector and Rice Transformation

A single guide RNA (sgRNA) sequence targeted the receiver-domain region of the *Ehd1* gene were designed according to Shan’s program (Shan et al., 2014). A sgRNA-Cas9 vector pVK005-01 (Viewsolid Biotech, Beijing, China) in which a codon-optimized *Cas9* gene was under control of the maize ubiquitin promoter and the customized sgRNA was driven by the rice *U6* promoter, was used for generation of the sgRNA-Cas9 construct for *Ehd1*. DNA oligos (Supplementary Table S1) corresponding to the designed sgRNA sequence were synthesized and the dimer was cloned into pVK005-01 according to the manufacturer’s instruction.

The *Ehd1* sgRNA-Cas9 expression vector was introduced into *Agrobacterium tumefaciens* strain EHA105 by electroporation. *Agrobacterium*-mediated transformation of rice calli induced from seeds of Nipponbare, Longdao16, Longdao24, and Xiushui134 was carried out as described previously (Hiei et al., 1997).

### Genotyping and Off-Target Analysis

Genomic DNA was extracted from rice leaves using the CTAB method. The T_0_ transgenic plants were determined by PCR amplification of the *hygromycin phosphotransferase* gene. To genotype mutations in the *Ehd1* gene, PCR amplification was performed using primer pairs flanking the *Ehd1* target site (Supplementary Table S1). The PCR products were sequenced by Sanger method. DNA sequences were aligned using Clustal Omega (Sievers et al., 2011).

Transgene-free homozygous mutant plants/lines were screened in T_1_ generation derived from self-pollination of T_0_ mutant plants, and were double confirmed in T_2_ generation. To identify transgene-free plants, PCR amplification was performed with DNA extracted from individual plants using specific primers (Supplementary Table S1) for the *hygromycin phosphotransferase* gene and the *Cas9* gene.

Forty transgene-free T_2_ mutant plants in Nipponbare, Longdao16, Longdao24, and Xiushui134 backgrounds were selected for evaluation of the potential off-target effects. PCR amplification was performed using specific primers (Supplementary Table S1) for the potential off-target sites. The resulting PCR products were sequenced for mutations by Sanger method.

### Plant Growth Conditions and Phenotype Analysis

T_0_ generation plants were grown in a standard greenhouse. Wild-type parents, T_1_ and T_2_ transgene-free homozygous mutant plants were investigated for agronomic traits. The plants were cultivated at Sanya experimental station, Hainan Province, under nature SD condition (December to next April, c. 11–12.5 h, 18°14′N, 109°31′E), and at Fuzhou experimental station, Fujian Province, under nature LD condition (mid-May to mid-September, c. 13.5 h, 26°08′N, 119°28′E). Longdao16 and Longdao24 were also cultivated at Haerbing experimental station, Heilongjiang Province, under nature LD condition (April to September, c. 13.5–15.7 h, 45°51′N, 126°50′E). About 100 seedlings of each line were transplanted in the field. Column space and row were both 20 cm. The field management was followed the normal agricultural practice. Head rice rate was determined by the percentage of the total head rice weight produced after milling. Grain chalkiness traits were determined using image analysis method (Fang et al., 2015), and grain amylose content was determined by iodine reaction (Juliano, 1971). Gel consistency and alkali spreading value were determined as described previously (Leng et al., 2013).

One T_2_ Nipponbare mutant line N-ehd1-#1, and two T_2_ Longdao16 mutant lines L16-end1-#2, L16-end1-#3 were used for analyzing gene expression of *Hd3a* and *RFT1* at different developmental stages. The frame-shift mutant lines N-ehd1-#2, L16-end1-#1, and in-frame mutant line L16-end1-#6 were also analyzed for the mRNA expression levels of *Ehd1, Hd3a*, and *RFT1* at 50d after sowing. All the lines were grown under LD condition (mid-May to mid-August, c. 13.5 h, before heading) in nature filed and SD condition (September to next January, c. 12.7–11.5 h) in a greenhouse at Fuzhou experimental station. Full expanded leaves were sampled 2 h after dawn. Three independent plants were used as biological replicates.

### Gene Expression Analysis

Total RNAs were isolated from rice leaves using RNA isolater Total RNA Extraction Reagent (Vazyme Biotech Co., Ltd., Nanjing, China) according to the manufacturer’s instructions. The RNA samples were treated with Recombinant DNase I (TaKaRa, Shiga, Japan). First cDNA was synthesized with 2 μg of total RNA using a RevertAid First Strand cDNA Synthesis Kit (Thermo Fisher Scientific, UC, Lithuania). The synthesized cDNAs were used as templates for quantitative real-time RT-PCR with ChamQ^TM^ SYBR qPCR Master Mix (Vazyme Biotech, Nanjing, China) on a QuantStudio 3 Real-Time PCR System (Thermo Fisher Scientific, Singapore). All experiments were conducted three times. The primers used for gene expression analysis are listed in Supplementary Table S1.

## Results

### Site-Specific Mutagenesis of *Ehd1* Mediated by CRISPR/Cas9 System

**FIGURE 1:**
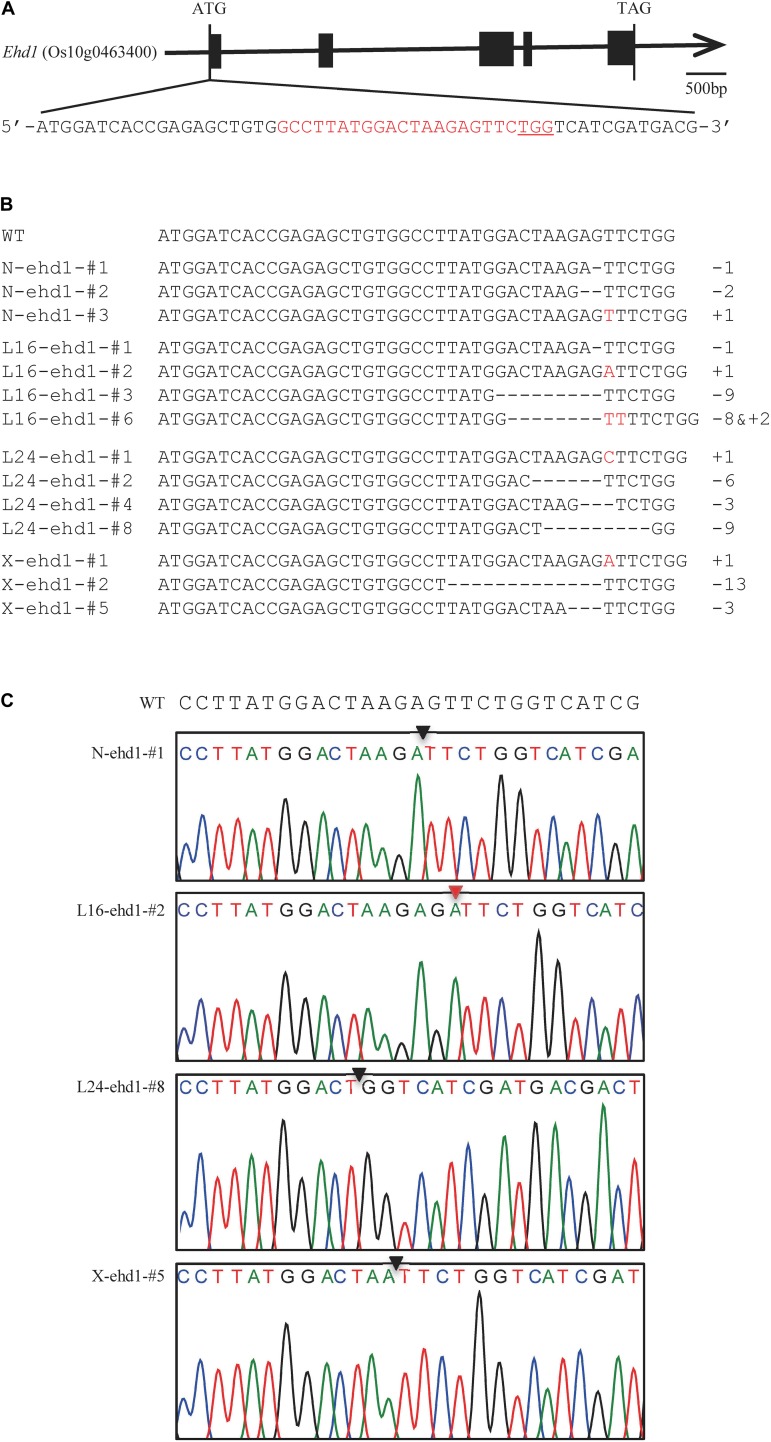
CRISPR/Cas9-mediated genome editing of the *Ehd1* gene. **(A)** Schematic illustration of the *Ehd1* gene structure. Black rectangles represent the 5 exons of *Ehd1*; Red characters indicate the sequence of the target site; PAM sequences are underlined. **(B)** Examples of mutations of *Ehd1* in CRISPR/Cas9-mediated mutated plants. Deletions and insertions are indicated by dashes and red letters, respectively, Numbers on the right side indicate the numbers of nucleotides involved (+, insertion; −, deletion); WT indicates wild-type sequence; N-ehd1-#, L16-ehd1-#, L24-ehd1-#, and X-ehd1-# represent the mutant plants in Nipponbare, Longdao16, Longdao24, and Xiushui134 backgrounds, respectively. **(C)** Examples of sequencing chromatograms of an 1 bp deletion mutant (N-ehd1-#1), an 1 bp insertion mutant (L16-ehd1-#2), a 9 bp deletion mutant (L24-ehd1-#8), and a 3 bp deletion mutant (X-ehd1-#5), in Nipponbare, Longdao16, Longdao24, and Xiushui134 backgrounds, respectively. The deletion and insertion sites are indicated by the black triangle and red triangle, respectively.

To generate transgenic plants with targeted mutation in *Ehd1*, a sgRNA-Cas9 construct targeting the first exon of the *Ehd1* gene (Figure 1A) was designed and used for rice transformation. A total of 30, 28, 25, and 20 T_0_ independent transgenic plants were generated from calli of *japonica* rice varieties Nipponbare, Longdao16, Longdao24, and Xiushui134, respectively (Table 1 and Supplementary Table S2). Genotyping of the transgenic plants identified 53 (14, 15, 13, and 11 in Nipponbare, Longdao16, Longdao24, and Xiushui134 backgrounds, respectively) with mutations in the predicted target site of *Ehd1* (Figure 1, Table 1, and Supplementary Table S2). The mutations in T_0_ transgenic plants included homozygous, bi-allelic and heterozygous genotypes (Table 1 and Supplementary Table S2). Most of the mutations were frame-shift types in mutants in Nipponbare, Longdao16, Longdao24, and Xiushui134 backgrounds. In addition, there were two, three, and one mutants with in-frame deletion in the target site in Longdao16, Longdao24, and Xiushui134 backgrounds, respectively (Figure 1B and Supplementary Table S2). The mutants in Nipponbare, Longdao16, Longdao24, and Xiushui134 backgrounds were thus referred to as N-ehd1, L16-ehd1, L24-ehd1, and X-ehd1, respectively.

**TABLE 1:** Mutation analysis of T_0_ plants transformed with the sgRNA-Cas9 construct targeting the first exon of the *Ehd1* gene.

Variety	No. of transgenic plants	No. of plants with mutations (%)*	T_0_ zygosity			Mutation type	
			Homozygous (%)*	Bi-allele (%)*	Heterozygous (%)*	Frame-shift (%)†	In-frame deletion (%)^†^
Nipponbare	30	14 (46.67)	3 (10.00)	4 (13.33)	7 (23.33)	14 (100.00)	0 (0.00)
Longdao16	28	15 (53.57)	4 (14.29)	3 (10.71)	8 (28.57)	13 (86.67)	2 (13.33)
Longdao24	25	13 (52.00)	2 (8.00)	3 (12.00)	8 (32.00)	10 (76.92)	3 (23.08)
Xiushui134	20	11 (55.00)	2 (10.00)	1 (5.00)	8 (40.00)	10 (90.91)	1 (9.09)

*Percentages were calculated based on the number of plants with mutations over the total number of transgenic plants.^†^Percentages were calculated based on the number of plants with frame-shift or in-frame deletion mutations over the number of plants with mutations.

Transgene-free T_1_ and T_2_ mutant lines were screened (Supplementary Figures S1, S2) and T_2_ mutant lines were examined for potential off-target mutations. Six potential off-target sites containing four or five mismatched bases were retrieved using the CRISPR-P 2.0 tool (Liu et al., 2017). PCR products amplified from 40 transgene-free T_2_ mutant plants (9, 11, 10, and 10 in Nipponbare, Longdao16, Longdao24, and Xiushui134 backgrounds, respectively) were sequenced. No mutations were found in all six potential sites (Supplementary Table S3), indicating the high specificity of the designed sgRNA in mediating mutagenesis of the predicted site.

### Frame-Shift Mutations in *Ehd1* Resulted in Long BVG Periods in Nipponbare, Longdao16, Longdao24, and Xiushui134

**FIGURE 2:**
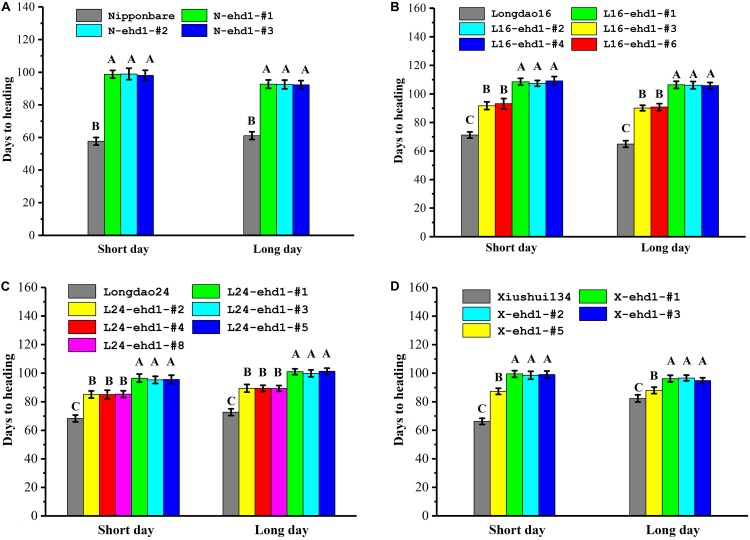
*ehd1* mutant lines exhibited prolonged basic vegetative growth (BVG) periods under short day and long day conditions. Rice plants were cultivated at Sanya experimental station under nature short day condition (December to next April, c. 11–12.5 h, 18°14′ N, 109°31′E), and at Fuzhou experimental station under natural long day condition (mid-May to mid-September, c. 13.5 h, 26°08′N, 119°28′E). **(A–D)** Flowering times of homozygous mutant lines in Nipponbare **(A)**, Longdao16 **(B)**, Longdao24 **(C)** and Xiushui134 **(D)** backgrounds under both short day and long day conditions. Data were collected from 30 plants. The letters A, B, and C indicate significant differences according to LSD multiple range test at *P* ≤ 0.01.

Transgene-free homozygous T_1_ and T_2_ mutant lines were investigated for heading date under nature SD (Sanya, December to next April, c. 11–12.5 h, 18°14′N, 109°31′E) and LD (Fuzhou, mid-May to mid-September, c. 13.5 h, 26°08′N, 119°28′E) environment conditions in low-latitude regions. As showed in Figure 2 and Supplementary Figure S3, the flowering times of the frame-shift mutants were significantly delayed compared with that of wild-type in Nipponbare, Longdao16, Longdao24, and Xiushui134 backgrounds. For example, the heading dates of N-ehd1-#1, an 1-bp deletion homozygous mutant line in Nipponbare background (Figure 2A), were 98.1 ± 3.1 days under SD and 92.3 ± 2.5 days under LD, respectively. In contrast, the heading dates of wild-type Nipponbare were about 57.7 ± 2.3 days and 61.1 ± 2.3 days under SD and LD, respectively (Figure 2A). The heading dates of L16-ehd1-#1, an 1-bp deletion homozygous mutant line in Longdao16 background, were 108.5 ± 2.4 days under SD and 106.4 ± 2.5 days under LD, which were about 40 days more than that of wild-type Longdao16 plants under SD (71.2 ± 2.2 days) and LD (64.9 ± 2.3 days), respectively (Figure 2B). Similar results were observed in other tested frame-shift mutant lines in all four backgrounds (Figure 2), indicating that frame-shift mutations in *Ehd1* could result in long BVG periods in *japonica* varieties under low-latitude conditions.

### In-Frame Deletion Mutations in the Receiver Domain Region of Ehd1 Resulted in Moderate Delays of Heading Date in Longdao16, Longdao24, and Xiushui134

**FIGURE 3:**
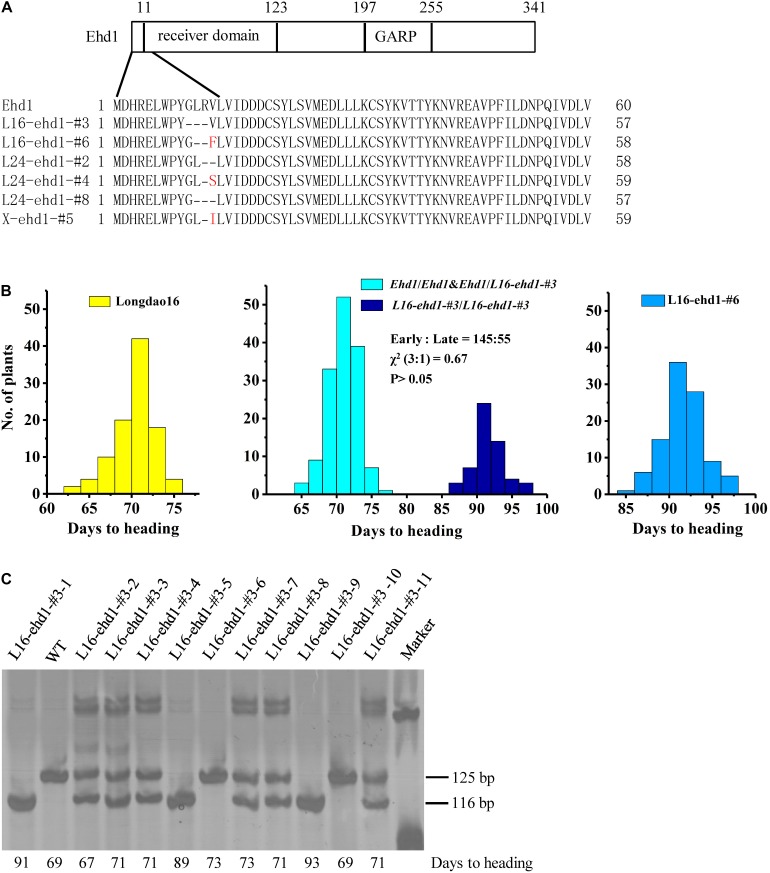
Minor in-frame deletion mutations in the receiver domain region of Ehd1 caused moderate delays of heading date in rice. **(A)** Schematic diagram of the domains within Ehd1 and comparison of deduced amino acid sequences of Ehd1 in the six in-frame deletion mutant lines L16-ehd1-#3, L16-ehd1-#6, L24-ehd1-#2, L24-ehd1-#4, L24-ehd1-#8, and X-ehd1-#5. Deletion and substitution are indicated by dash and red letter, respectively. **(B)** Frequency distributions of days to heading of Longdao16 (left), T_1_ segregating population of L16-ehd1-#3 (middle), and T_1_ homozygous line L16-ehd1-#6 (right). About 100 plants, 200 plants and 100 plants of Longdao16, L16-ehd1-#3, and L16-ehd1-#6, respectively, were investigated. **(C)** Examples of genotyping and phenotyping results showing co-segregation of the moderately delayed flowering phenotype with the homozygous *L16-ehd1-#3* allele in T_1_ population of L16-ehd1-#3. Each individual plant was analyzed by PCR amplification using allele-specific primers. The 125 and 116 bp fragments represent wild-type *Ehd1* and *L16-ehd1-#3* alleles, respectively. Days to heading of each detected plant are shown at the bottom.

Six in-frame deletion lines, L16-ehd1-#3, L16-ehd1-#6, L24-ehd1-#2, L24-ehd1-#4, L24-ehd1-#8, and X-ehd1-#5, were identified in Longdao16, Longdao24, or Xiushui134 backgrounds, respectively. Sequencing results of the mutated target site showed that the change numbers of nucleotides were multiples of three in the six lines (Figure 3 and Supplementary Figure S4). For example, L16-ehd1-#3 was a mutant with 9-bp deletion (5′-GACTAAGAG-3′) (Supplementary Figure S4) and L16-ehd1-#6 was a mutant with 8 bp deletion and 2 bp insertion (5′-ACTAAGAGTT-3′) (Supplementary Figure S4). To examine whether the mutated alleles had different splicing patterns of *ehd1*, RT-PCR was performed to amplify the open reading frame (ORF) of the mutated *ehd1* in all six lines. Sequencing results revealed that the splicing pattern of these six novel alleles was unchanged (Supplementary Figure S4). Prediction from the mutated *ehd1-ORF* sequences revealed that there were minor deletions (two- to three-amino acid) in the receiver domain of ehd1 in L16-ehd1-#3, L24-ehd1-#2, and L24-ehd1-#8, and there were minor deletions (one- to two-amino acid) plus a Val-to-Phe, -Ser or -Ile substitution in L16-ehd1-#6, L24-ehd1-#4, and X-ehd1-#5 (Figure 3A).

Under both SD and LD, the flowering times of the homozygous in-frame deletion plants in Longdao16 and Longdao24 backgrounds were delayed about 2–3 weeks compared with that of their wild-type parents, but were about 10 days to 2 weeks earlier than that of the frame-shift mutant lines in the same backgrounds (Figures 2B,C). Similar results were observed in the in-frame deletion line X-ehd1-#5 in Xiushui134 background, although the delay of flowering time under LD was not as great as that under SD (Figure 2D). Overall, the in-frame deletion mutant lines exhibited moderate delays of heading date. To further confirm the correlation between the in-frame deletion *ehd1* alleles and the moderately-delayed flowering phenotype, a total of 200 segregating T_1_ progeny derived from T_0_ L16-ehd1-#3 were phenotyped and genotyped individually. The T_1_ L16-ehd1-#3 plants exhibited a segregation of 145 early flowering and 55 moderately-delayed flowering, fitting to a 3:1 ratio (χ^2^ = 0.67, *P* > 0.05) (Figure 3B). The genotyping result showed that the segregating ratios of wild-type (*Ehd1/Ehd1*): heterozygous type (*Ehd1/L16-ehd1-#3*): homozygous type (*L16-ehd1-#3/L16-ehd1-#3*) fitted the 1:2:1 ratio of segregation (42:103:55, χ^2^ = 0.87, *P* > 0.05). More importantly, the homozygous *L16-ehd1-#3* allele was co-segregated with the moderately-delayed flowering phenotype (Figures 3B,C).

### In-Frame Deletion *ehd1* Allele Had a Moderate Effect on Activation of *Hd3a* and *RFT1*

The mRNA expression levels of *Ehd1*, *Hd3a* and *RFT1* in homozygous T_2_ lines N-ehd1-#1, N-ehd1-#2, L16-end1-#1, L16-ehd1-#2, L16-ehd1-#3, and L16-end1-#6 under both SD and LD were detected. The results showed no significant difference in the mRNA levels of *Ehd1* between Nipponbare and Longdao16 under SD. In contrast, *Ehd1* mRNA level in Nipponbare was significantly lower than that in Longdao16 under LD (Supplementary Figure S5). In Nipponbare background, there was no significant difference in *Ehd1* mRNA levels between N-ehd1-#1, N-ehd1-#2, and wild-type plants. In Longdao16 background, *Ehd1* mRNA levels in the frame-shift mutant lines L16-end1-#1 and L16-ehd1-#2 were higher than that in wild-type plants or the in-frame deletion lines L16-ehd1-#3 and L16-end1-#6 (Supplementary Figure S5). These results suggested that mutagenesis mediated by CRISPR/Cas9 affected Ehd1 at the protein level but not the transcript level.

**FIGURE 4:**
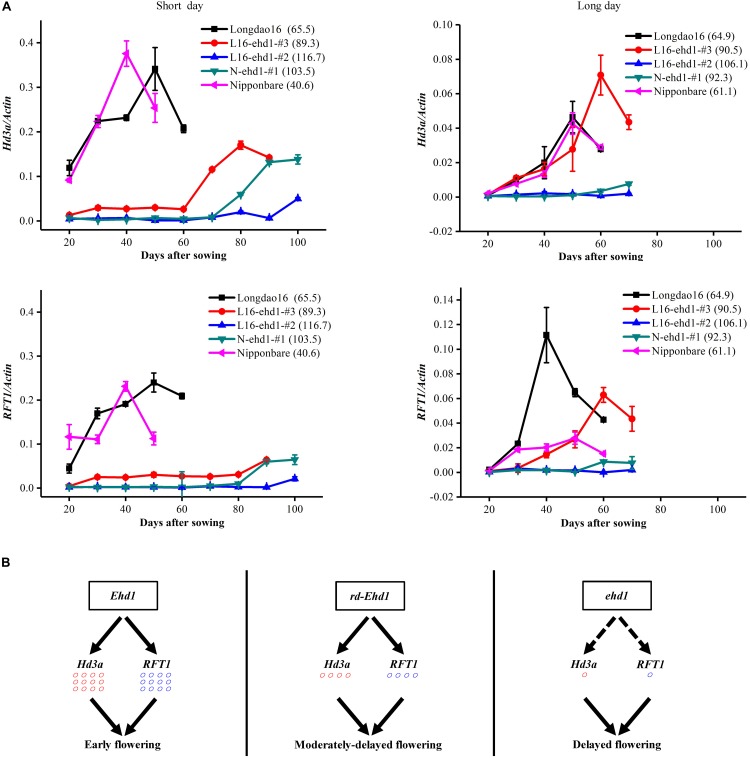
Minor in-frame deletion in the receiver domain partially impairs the function of Ehd1 on activation of *Hd3a* and *RFT1*. **(A)** Expression patterns of *Hd3a* and *RFT1* in frame-shift mutant lines N-ehd1-#1 and L16-end1-#2, and in-frame mutant line L16-end1-#3. T_2_ homozygous lines were used for detection. Fully expanded leaves were sampled 2 h after dawn every 10 days (the first time to harvest samples was 20 days after sowing). Three different rice plants were used as biological replicates and the experiments were repeated three times with similar results. Gene expression values are shown as mean and standard deviation of the three biological replicates. Days to heading of each line are shown in parentheses. **(B)** Schematic model of the minor deletion*-ehd1-Hd3a/RFT1* regulatory network. Functional *Ehd1* promotes the expression of two florigen genes *Hd3a* and *RFT1* (left panel). In *ehd1* background, the expression of *Hd3a* and *RFT1* was inhibited (right panel). In-frame deletion *ehd1* partially activates the expression of *Hd3a* and *RFT1* (middle panel), resulting in a moderately-delayed flowering phenotype. *rd-Ehd1* represents in-frame deletion *ehd1* alleles.

During the vegetative growth phrase, *Hd3a* and *RFT1* were expressed at relatively high levels in wild-type varieties Nipponbare and Longdao16. In contrast, *Hd3a* and *RFT1* were expressed at low levels in the frame-shift mutant lines N-ehd1-#1, N-ehd1-#2, L16-end1-#1, and L16-ehd1-#2 (Figure 4A and Supplementary Figure S6). The expression levels of *Hd3a* and *RFT1* in the in-frame deletion lines L16-ehd1-#3 and L16-end1-#6 were lower than that in wild-type Longdao16, but were higher than that in L16-end1-#1 and L16-ehd1-#2 (Figure 4A and Supplementary Figure S6), although expression patterns were not exactly the same under SD and LD, respectively. We further performed the liner fitting analysis of the relationship between heading date and expression levels of *Hd3a* and *RFT1* in L16-ehd1-#2, L16-ehd1-#3, and Longdao16. The analysis revealed that there was a significantly negative correlation between heading date and expression levels of *Hd3a* and *RFT1* (Supplementary Figure S7). These results thus supported the conclusion that transcriptional activation of *Hd3a* and *RFT1* mainly relied on the presence of functional Ehd1 (Doi et al., 2004; Zhao et al., 2015; Cho et al., 2016; Nemoto et al., 2016). Our results also indicated that minor deletions in the receiver domain could quantitatively impair the function of Ehd1 on activation of *Hd3a* and *RFT1*, resulting in a moderately-delayed flowering phenotype (Figure 4B).

### In-Frame and Fame-Shift Mutant Lines Exhibited High Grain Yield and Good Grain Quality in Low-Latitude Regions

**FIGURE 5:**
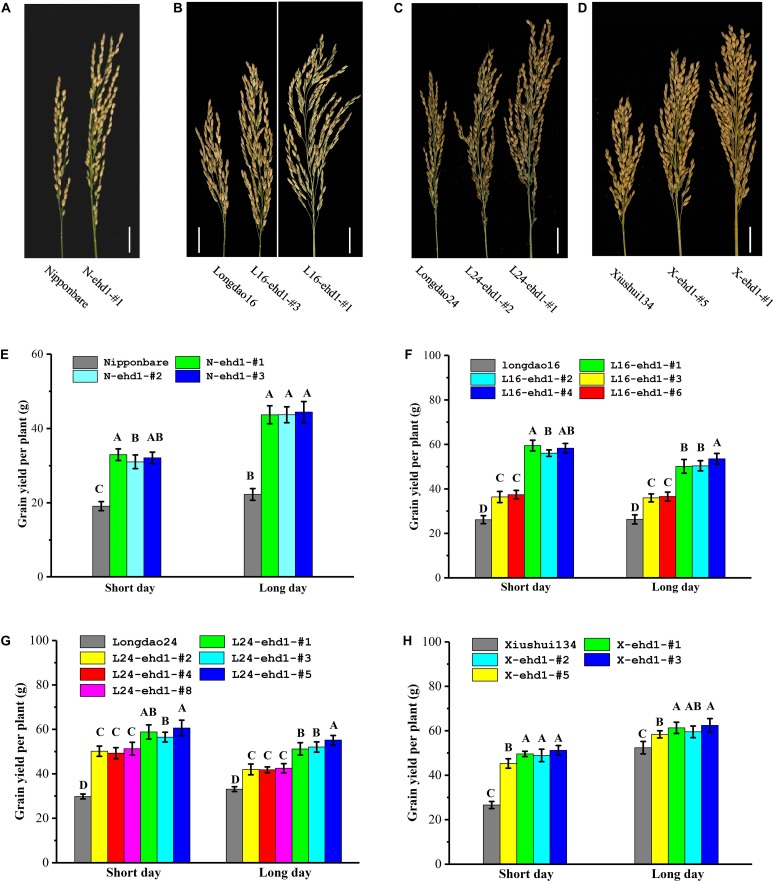
*ehd1* mutant lines exhibited high grain yield under both LD and SD conditions. **(A–D)** *ehd1* mutant lines exhibited significantly increased panicle length and grain number per panicle in Nipponbare **(A)**, Longdao16 **(B)**, Longdao24 **(C)**, and Xiushui134 **(D)** backgrounds, respectively. Pictures were taken at Fuzhou experimental station under long day condition. Scale bar: 2 cm. **(E–H)** Statistical analysis for grain yield per plant of *ehd1* mutant lines in Nipponbare **(E)**, Longdao16 **(F)**, Longdao24 **(G)**, and Xiushui134 **(H)** backgrounds. Data were collected from 10 plants. The letters A, B, C, and D indicates the significant differences according to LSD multiple range test at *P* ≤ 0.01.

Homozygous in-frame and frame-shift mutant lines were investigated under both SD and LD in low-latitude regions for yield-associated traits, including plant height, panicle number per plant, panicle length, 1000-grains weight, spikelets per panicle, and grain yield per plant. Due to the prolongation of growth period, both the in-frame and frame-shift mutant lines exhibited significantly higher plant height, panicle length, especially spikelets per panicle, which in turn resulted in higher grain yield per plant compared with wild-types (Figure 5 and Table 2). For example, the grain yields per plant of the in-frame mutant lines L16-ehd1-#3 and L16-ehd1-#6 were about 1.37- to 1.43-fold, and the grain yields per plant of the frame-shift lines L16-ehd1-#1, L16-ehd1-#2, and L16-ehd1-#4 were about 1.90–2.28-fold compared with that in wild-type Longdao16 plants under SD or LD (Figure 5F). Similar results were observed in other homozygous frame-shift lines in Nipponbare, Longdao24, or Xiushui134 backgrounds (Figures 5E,G,H).

**TABLE 2:** Major agronomic traits of the *ehd1* mutant lines under both SD and LD.

Condition*	Rice line	Plant height (cm)^†^	Panicle number per plant^†^	Panicle length (cm)^‡^	Spikelets per panicle^‡^	1,000-grain weight (g)^§^
SD	Nipponbare	56.2 ± 2.5D	21.8 ± 1.9a	12.1 ± 1.0B	36.7 ± 6.1C	25.4 ± 0.2a
	N-ehd1-#1	83.2 ± 3.6B	21.2 ± 1.9a	17.8 ± 2.3a	67.7 ± 12.1B	25.2 ± 0.2a
	N-ehd1-#2	79.2 ± 2.6C	22.0 ± 1.6a	17.6 ± 1.9a	72.5 ± 10.8A	25.5 ± 0.4a
	N-ehd1-#3	85.6 ± 3.5A	20.6 ± 2.1a	17.5 ± 2.2A	73.6 ± 15.1A	25.3 ± 0.2a
	Longdao16	102.8 ± 4.8C	12.6 ± 3.2D	18.5 ± 1.8C	99.5 ± 27.1C	24.5 ± 0.4a
	L16-ehd1-#1	120.3 ± 3.2A	17.5 ± 1.3A	22.9 ± 1.8A	170.9 ± 35.0A	24.4 ± 0.3a
	L16-ehd1-#2	120.8 ± 1.5A	16.8 ± 1.8B	23.3 ± 2.7A	164.3 ± 16.3A	24.4 ± 0.4a
	L16-ehd1-#3	110.5 ± 2.7B	15.3 ± 1.9C	20.3 ± 1.9B	118.2 ± 22.6B	24.6 ± 0.4a
	L16-ehd1-#4	119.6 ± 3.2A	17.2 ± 1.5A	23.0 ± 2.4A	168.5 ± 22.3A	24.6 ± 0.3a
	L16-ehd1-#6	111.5 ± 3.1B	15.5 ± 2.1C	20.5 ± 1.6B	122.6 ± 19.8B	24.5 ± 0.4a
	Longdao24	90.3 ± 1.7C	8.8 ± 0.4b	18.3 ± 1.9D	155.6 ± 46.8C	24.0 ± 0.2a
	L24-ehd1-#1	120.3 ± 2.9A	10.2 ± 1.9a	23.9 ± 1.6B	275.7 ± 28.4A	23.9 ± 0.2a
	L24-ehd1-#2	104.4 ± 2.1B	10.8 ± 1.5a	22.3 ± 0.9C	210.0 ± 22.4B	23.8 ± 0.1a
	L24-ehd1-#3	118.8 ± 2.4A	10.1 ± 2.2a	24.6 ± 1.2A	284.2 ± 44.6A	23.9 ± 0.1a
	L24-ehd1-#4	104.1 ± 1.1B	10.0 ± 1.3a	22.7 ± 1.5C	227.8 ± 36.5B	24.1 ± 0.3a
	L24-ehd1-#5	117.8 ± 1.9A	11.3 ± 1.4a	25.0 ± 1.1A	285.7 ± 28.4A	24.0 ± 0.1a
	L24-ehd1-#8	103.3 ± 1.1B	11.0 ± 2.1a	22.3 ± 1.9C	216.7 ± 27.5B	23.9 ± 0.1a
	Xiushui134	63.4 ± 2.0C	11.6 ± 1.3a	14.0 ± 1.2C	94.4 ± 24.2D	26.2 ± 0.2a
	X-ehd1-#1	73.5 ± 2.4A	12.5 ± 1.5a	16.3 ± 0.9A	167.8 ± 20.4B	26.1 ± 0.2a
	X-ehd1-#2	73.8 ± 1.9A	12.2 ± 1.8a	16.6 ± 0.8A	174.7 ± 23.3AB	26.3 ± 0.3a
	X-ehd1-#3	72.8 ± 1.5A	11.8 ± 1.9a	16.4 ± 0.9A	181.8 ± 23.2A	26.2 ± 0.3a
	X-ehd1-#5	69.6 ± 2.9B	11.6 ± 0.8a	15.3 ± 0.9B	156.0 ± 30.2C	26.3 ± 0.4a
LD	Nipponbare	62.2 ± 1.9D	20.6 ± 3.1a	16.6 ± 1.1C	53.2 ± 9.6C	24.5 ± 0.1a
	N-ehd1-#1	90.8 ± 1.1A	18.8 ± 1.1a	20.1 ± 2.3A	113.5 ± 22.8A	24.4 ± 0.3a
	N-ehd1-#2	86.2 ± 0.8C	20.4 ± 3.6a	18.8 ± 1.5AB	103.8 ± 17.8B	24.6 ± 0.2a
	N-ehd1-#3	89.0 ± 1.4B	22.0 ± 4.8a	18.6 ± 1.8B	108.1 ± 22.9AB	24.7 ± 0.3a
	Longdao16	90.4 ± 3.7D	12.0 ± 1.6ab	20.0 ± 1.7C	114.4 ± 21.1D	23.6 ± 0.3a
	L16-ehd1-#1	121.6 ± 1.5B	13.0 ± 2.2a	23.7 ± 1.8A	198.6 ± 31.3B	23.6 ± 0.3a
	L16-ehd1-#2	126.8 ± 2.2A	12.6 ± 1.8a	23.8 ± 1.8A	211.7 ± 45.5A	23.7 ± 0.2a
	L16-ehd1-#3	109.4 ± 0.6C	12.2 ± 1.3ab	21.7 ± 1.6B	151.7 ± 21.0C	23.7 ± 0.4a
	L16-ehd1-#4	128.0 ± 2.1A	10.8 ± 1.8b	23.7 ± 2.2A	218.1 ± 34.4A	24.2 ± 0.5a
	L16-ehd1-#6	109.4 ± 2.1C	12.0 ± 0.7ab	21.7 ± 1.0B	158.6 ± 20.6C	23.9 ± 0.4a
	Longdao24	88.1 ± 3.8C	10.8 ± 1.2a	20.3 ± 2.1D	141.4 ± 29.2C	23.6 ± 0.2a
	L24-ehd1-#1	115.7 ± 1.8A	12.6 ± 2.9a	24.4 ± 2.2B	200.7 ± 40.7A	23.5 ± 0.2a
	L24-ehd1-#2	105.0 ± 4.4B	10.9 ± 2.5a	22.4 ± 1.6C	176.9 ± 34.3B	23.7 ± 0.3a
	L24-ehd1-#3	117.3 ± 4.7A	12.4 ± 3.5a	26.4 ± 2.1A	207.3 ± 44.6A	23.6 ± 0.2a
	L24-ehd1-#4	102.9 ± 1.9B	11.1 ± 3.1a	22.7 ± 1.9C	174.3 ± 23.0B	23.5 ± 0.3a
	L24-ehd1-#5	116.3 ± 2.0A	12.8 ± 2.3a	26.7 ± 1.8A	219.1 ± 30.9A	23.7 ± 0.3a
	L24-ehd1-#8	101.3 ± 2.7B	12.7 ± 3.2a	23.3 ± 1.7BC	169.5 ± 25.6B	23.6 ± 0.3a
	Xiushui134	80.7 ± 2.6C	12.5 ± 1.3a	16.2 ± 1.1c	181.0 ± 18.4C	26.0 ± 0.2a
	X-ehd1-#1	89.5 ± 2.6A	13.3 ± 0.9a	18.1 ± 1.2a	227.7 ± 39.1A	25.8 ± 0.2a
	X-ehd1-#2	86.9 ± 1.9A	13.1 ± 1.1a	17.6 ± 1.0ab	220.2 ± 21.0A	25.9 ± 0.3a
	X-ehd1-#3	87.7 ± 1.8A	13.7 ± 1.8a	17.9 ± 1.2a	231.2 ± 33.5A	26.1 ± 0.3a
	X-ehd1-#5	84.0 ± 1.6B	13.0 ± 1.5a	16.8 ± 0.9b	202.3 ± 22.2B	25.9 ± 0.2a

*Rice plants were cultivated at Sanya experimental station under natural SD condition (December to next April, c. 11–12.5 h, 18°14′N, 109°31′E), and at Fuzhou experimental station under natural long day condition (mid-May to mid-September, c. 13.5 h, 26°08′N, 119°28′E).^†^Plant height and panicle number per plant were collected from 10 plants.^‡^Panicle length and spikelets per plant were collected from 40 panicles.^§^ 1000-grain weight was collected from 3000 seeds.A, B, C, and D indicate significant differences according to LSD multiple range test at P ≤ 0.01, and a and b indicate significant differences at P ≤ 0.05.

Rice grains harvested from wild-types and the *ehd1* mutant lines cultivated in Fuzhou, and from the two Heilongjiang varieties Longdao16 and Longdao24 cultivated in Haerbing, Heilongjiang, were evaluated for quality characteristics, including head rice rate, chalkiness degree, chalky rice rate, amylose content, gel consistency, and alkali spreading value. The results showed that, Longdao16 and Longdao24 cultivated in Heilongjiang had higher head rice rate, lower chalky rice rate and chalkiness degree compared with Longdao16, Longdao24 and the *ehd1* mutant lines cultivated in Fujian. There were no significant differences among the lines for other traits (Supplementary Figure S8). When compared with wild-types cultivated in Fuzhou, the in-frame and frame-shift *ehd1* mutant lines showed higher head rice rate, lower chalky rice rate and chalkiness degree (Supplementary Figure S8), which may be due to that the *ehd1* mutant lines were exposed to relatively lower temperature during grain-filling stage. There were no significant differences in amylose content, gel consistency and alkali spreading value between the *ehd1* mutant lines and their corresponding wild-types (Supplementary Figure S8). Overall, the *ehd1* lines exhibited high grain yield and good grain quality when cultivated in low-latitude regions.

## Discussion

### Rapid Breeding of New Elite *Japonica* Varieties With Prolonged BVG Periods and High Yield for Low-Latitude Regions Through Genome Editing of the *Ehd1* Gene

Previous studies revealed that grain yield of rice is positively correlated with growth period. A longer BVG period could promote the accumulation and allocation of more resources to reproductive organs, resulting in higher yield (Xue et al., 2008; Wei et al., 2010; Hu et al., 2013; Yan et al., 2013; Zhang et al., 2015; Leng et al., 2019). Severe shortening of growth period is considered to be the main obstacle for introducing northern *japonica* varieties to southern areas in China. Therefore, breeding of *japonica* varieties with long BVG periods would be of great significance to promote “*indica* to *japonica*” projects in southern China (Leng et al., 2019).

A growing number of studies showed that enhanced expression of or mutations in flowering time genes could lead to heading date changes in rice (Xue et al., 2008; Wei et al., 2010; Hu et al., 2013; Koo et al., 2013; Yan et al., 2013; Zhang et al., 2015; Wu et al., 2017; Leng et al., 2019). For example, enhanced expression of *Ghd7*, an upstream negative regulator of *Ehd1*, could lead to delayed heading in rice under LD. In contrast, natural mutants with reduced function of *Ghd7* exhibited early flowering (Xue et al., 2008). DTH7, DTH8 are two suppressors in the signal network of photoperiodic flowering. Expression of *DTH7* or *DTH8* led to delayed heading by down-regulating expression of *Ehd1* and *Hd3a* in rice under LD (Wei et al., 2010; Yan et al., 2013; Gao et al., 2014). Natural mutation of *DTH7* or *DTH8* caused weak photoperiod sensitivity. More recently, a preponderant allele of *Heading date 1* (*Hd1*) has been identified from *indica* cultivars, and backcrossing the preponderant *Hd1* allele led to prolongation of growth period of a *japonica* variety Chunjiang06 (Leng et al., 2019). These studies also demonstrated that, due to the prolongation of growth period, rice lines with enhanced expression of *Ghd7*, *DTH7*, *DTH8* or introgression of the preponderant *Hd1* allele possessed significantly increased grain yield (Xue et al., 2008; Wei et al., 2010; Yan et al., 2013; Gao et al., 2014; Zhang et al., 2015; Leng et al., 2019).

In this study, we aimed to breed *japonica* rice with prolonged growth periods under both SD and LD. While many rice flowering time genes, such as *Ghd7*, *DTH7* or *DTH8*, function only under LD, *Ehd1* functions to promote rice flowering under both SD and LD (Doi et al., 2004; Komiya et al., 2008). More recently, Cui et al. (2019) demonstrated that a single−nucleotide insertion in the third exon of the *Ehd1* gene resulted in delayed heading date of the *japonica* rice variety Sasanishiki. In the present study, we chose the first exon of *Ehd1* as a target for CRISPR/Cas9-mediated genome editing for regulating heading date in rice. We generated both frame-shift and/or in-frame deletion mutants in four *japonica* varieties, Nipponbare, Longdao16, Longdao24, and Xiushui134. Similar to previous studies (Li et al., 2016; Zhou et al., 2016; Macovei et al., 2018; Zhang J. et al., 2018), we were able to obtain transgene-free mutant lines by segregating away the integrated T-DNA in T_1_ generations (Supplementary Figure S2). Field investigation further showed that, in all four *japonica* variety backgrounds, the flowering time of in-frame and frame-shift *ehd1* mutant lines was significantly delayed compared with that of wild-type plants under both SD and LD. In the present study, we also observed that the delay of flowering times of the Xiushui134 mutant lines was not as great as that of the *ehd1* mutant lines in Nipponbare, Longdao16, or Longdao24 backgrounds, suggesting that the effects of Ehd1 on heading date varied under different genetic backgrounds.

Accordingly, the *ehd1* mutant lines displayed significantly improved yield potential (Figure 5). When planting as middle-season rice in mid-May at Fuzhou station, wild-type Nipponbare, Longdao16, Longdao24, and Xiushui134 flowered in late-July to early-August, and were exposed to high temperatures during grain-filling stage. In contrast, the in-frame and frame-shift *ehd1* mutant lines started to flower in late-August to early-September. While high temperature during grain-filling stage has been shown to lead to higher chalk values (Yamakawa et al., 2007), the delay of flowering time allowed the *ehd1* mutant lines to be exposed to relatively lower temperatures during grain-filling stage, resulting in higher head rice rate, lower chalky rice rate and chalkiness degree (Supplementary Figure S8). In addition, we did not observe obvious differences in stress tolerance, disease resistance or lodging resistance between the *ehd1* mutant lines and their wild-type parents while they were cultivated in the field at Sanya station and Fuzhou station over two generations (data not shown). Taken together, our study demonstrates an effective approach to rapid breeding of elite *japonica* varieties with prolonged BVG periods and high yield for low-latitude regions through genome editing of *Ehd1*.

### In-Frame Editing of *Ehd1* for Developing Rice With Intermediate-Long BVG Periods

*Japonica* varieties with extremely long BVG periods are expected to be suitable for cultivating in low-latitude regions. In production practice, however, rice varieties with various growth periods are required for cultivation in diverse areas or under different cropping seasons. For example, low-altitude and single-season cropping areas are more likely to grow varieties with long growth periods. In contrast, varieties with intermediate-long growth periods would be more applicable to planting in relatively high altitude areas or under double-season cropping system. In the present study, we found it interesting that the in-frame deletion *ehd1* lines in Longdao16, Longdao24, and Xiushui134 backgrounds, displayed a moderately-delayed flowering phenotype. The flowering time of the five in-frame deletion *ehd1* lines in Longdao16 and Longdao24 backgrounds was delayed about 3 weeks compared with that of wild-type plants, but was about 2 weeks earlier than that of the frame-shift lines (Figure 5). Similar moderately-delayed flowering phenotypes were observed in the in-frame deletion Xiushui134 mutant line X-ehd1-#5, although the delay of flowering time was less extreme. Ehd1 has a receiver domain and a GARP DNA-binding domain (Doi et al., 2004; Cho et al., 2016). In the receiver domain, the conserved Asp-Asp-Lys (D17th -D63th -K117th) motif, is known to be important for Ehd1 function (Takahashi et al., 2009; Cho et al., 2016). For example, phosphorylation of the middle Asp is required for homodimerization of Ehd1, which is crucial for Ehd1 activity (Cho et al., 2016). In the in-frame deletion *ehd1* lines, the editing caused minor deletion mutations of the first few residues of the Ehd1 receiver domain. These residues might not be critical for basic function of Ehd1. However, the moderately-delayed flowering phenotype of the six lines suggested that the minor in-frame mutations could quantitatively impair the biological function of Ehd1. Expression of *Hd3a* and *RFT1* in L16-ehd1-#3 was lower than that in wild-type Longdao16, but higher than that in the frame-shift line L16-ehd1-#2 (Figure 4), further supporting that the in-frame deletion *ehd1* allele had a moderate effect on activation of *Hd3a* and *RFT1*. Overall, our results showed that the in-frame deletion *ehd1* alleles have common effect among different backgrounds. Therefore, the in-frame deletion *ehd1* alleles could be valuable gene resources in developing rice varieties with intermediate-long BVG periods.

The majority of important agronomic traits in crops are quantitative traits. Thus, manipulation of quantitative traits has been extremely desired by breeders. Recently, CRISPR/Cas9-based *cis*-regulatory and upstream open reading frames (uORFs) mutagenesis approaches have been developed to create quantitative variations for crop breeding (Rodríguez-Leal et al., 2017; Zhang H. et al., 2018). Editing of the *cis*-regulatory elements or uORFs could cause transcriptional change of the targeted genes or the downstream primary ORFs (pORFs), leading to quantitative variation of the traits (Li et al., 2017; Zhang H. et al., 2018). Using the CRISPR/Cas9 system, Rodríguez-Leal et al. (2017) generated a continuum of variants of three major productivity traits (fruit size, inflorescence branching, and plant architecture) in tomato; Zhang H. et al. (2018) created mutant plant lines with varying amounts of mRNA translation in four pORFs. These results demonstrated the great feasibility of the two approaches for engineering quantitative traits for crop improvement. In the present study, we observed that minor in-frame deletion of non-critical residues of the Ehd1 receiver domain could result in quantitatively delayed flowering in rice. Our finding suggested that in-frame editing of non-critical residues could potentially be an alternative approach to creating quantitative variants of important traits. Unlike targeted editing in *cis*-regulatory regions that may change the expression patterns of the genes and cause unpredictable phenotypic variation (Rodríguez-Leal et al., 2017), in-frame editing does not affect expression pattern of the targeted genes, and may be applicable to traits controlled by specific expression patterns.

In summary, we report the development of *japonica* rice varieties with prolonged BVG periods and high yield potential by CRISPR/Cas9-mediated editing of *Ehd1*. Our results showed that, while frame-shift mutations in *Ehd1* resulted in more extremely long BVG periods, minor in-frame deletion mutations in the receiver domain region of Ehd1 led to moderate delays of heading date in *japonica* rice. Field investigation further showed that, both the in-frame and frame-shift lines exhibited significantly improved yield potential compared with wild-types. Therefore, our study demonstrated an effective approach to rapid breeding of elite *japonica* varieties with intermediate-long and more extremely long BVG periods. The *ehd1* mutant lines with the frame-shift or the novel in-frame deletion *ehd1* alleles represent valuable resources to develop elite *japonica* rice varieties for flexible cropping systems in diverse areas or under different seasons in southern China, and other low-latitude regions. In addition, our study suggests that in-frame editing of non-critical residues could potentially be an effective approach for engineering quantitative traits for crop improvement.

## Data Availability Statement

All datasets generated for this study are included in the article/Supplementary Material.

## Author Contributions

MW, SC, and FW conceived and designed the experiments. MW, HL, YL, JC, YF, JL, and ZZ performed the experiments. MW, KL, SC, and FW analyzed the data. MW, SC, and FW wrote the manuscript. All authors commented on the manuscript.

## Conflict of Interest

The authors declare that the research was conducted in the absence of any commercial or financial relationships that could be construed as a potential conflict of interest.
