# Supplementary material for: In-Frame and Frame-Shift Editing of the Ehd1 Gene to Develop Japonica Rice With Prolonged Basic Vegetative Growth Periods
Source: Front Plant Sci. 2020 Mar 19;11:307. doi: 10.3389/fpls.2020.00307 (PMC7096585; doi:10.3389/fpls.2020.00307)
Supplement: Supplementary file 1 [file Data_Sheet_1.PDF]

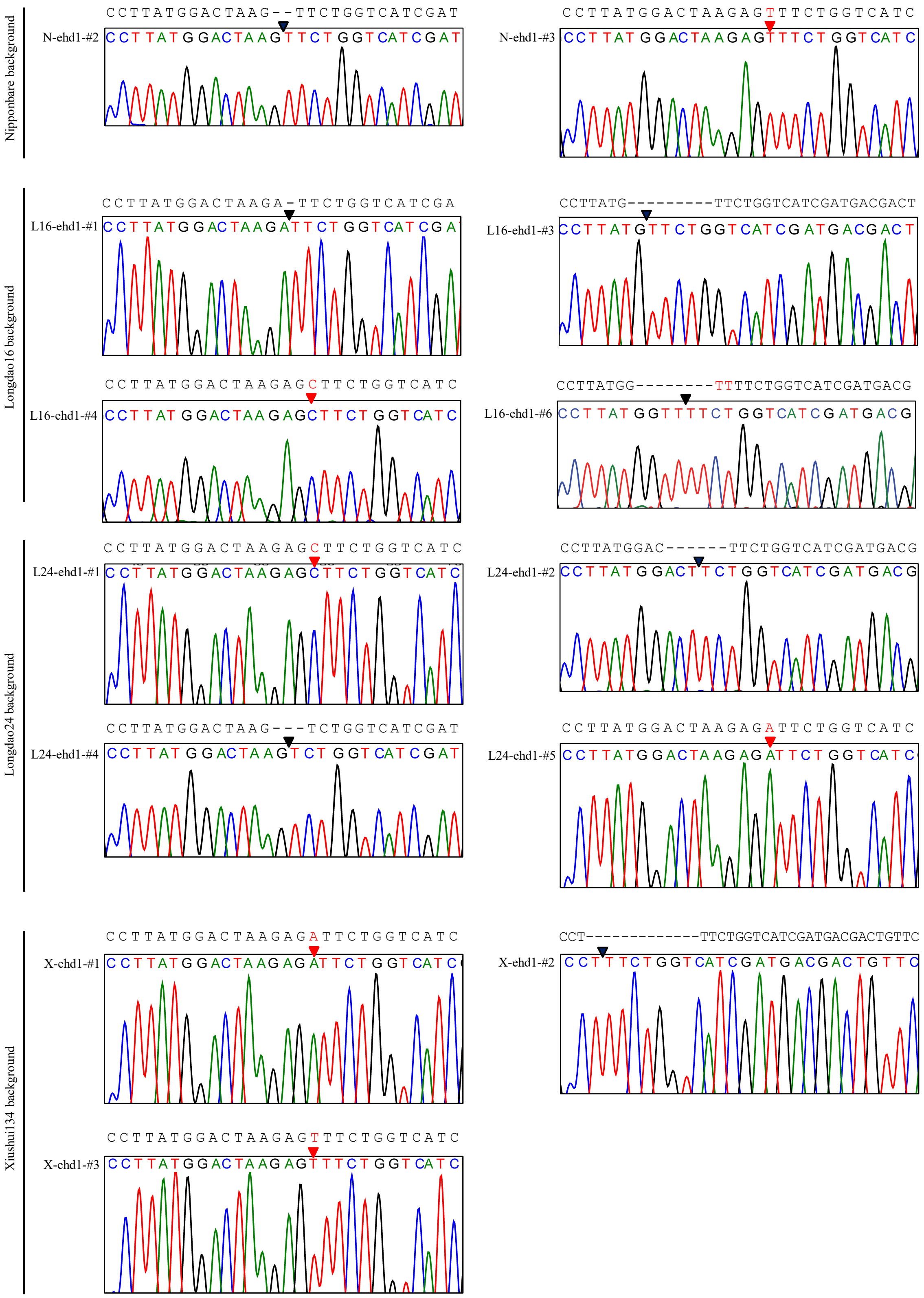

Supplementary Figure S1. Sequencing chromatograms of mutations in T_1 homozygous lines in Nipponbare, Longdao16, Longdao24, and Xiushui134 backgrounds. Deletions and insertions are indicated by dashes and red letters, respectively.
