# Supplementary material for: In-Frame and Frame-Shift Editing of the Ehd1 Gene to Develop Japonica Rice With Prolonged Basic Vegetative Growth Periods
Source: Front Plant Sci. 2020 Mar 19;11:307. doi: 10.3389/fpls.2020.00307 (PMC7096585; doi:10.3389/fpls.2020.00307)
Supplement: Supplementary file 2 [file Data_Sheet_2.PDF]

A

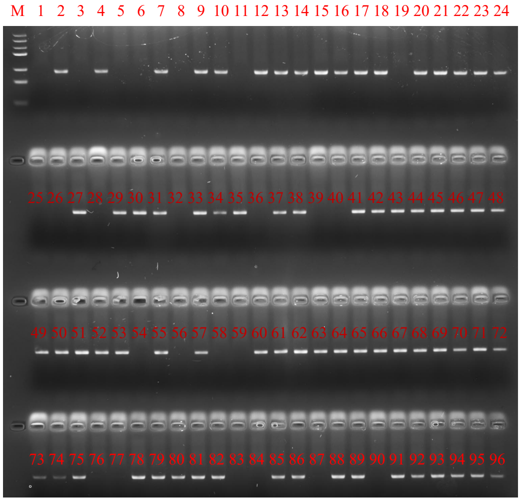

B

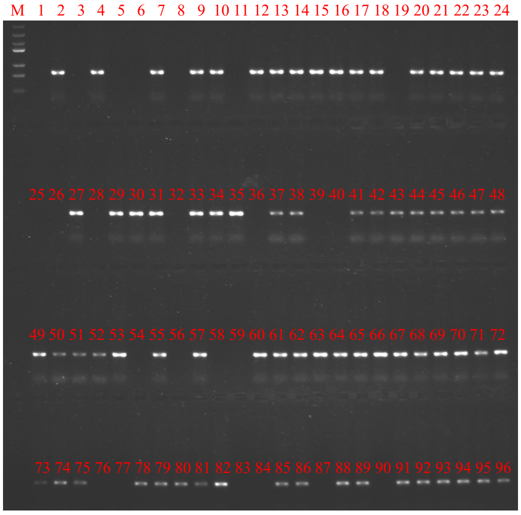

Supplementary Figure S2. PCR screening of transgene-free T_1 plants of L16-ehd1-#3. (A) PCR amplification for the *Hygromycin phosphotransferase* gene (*Hpt).* (B) PCR amplification for the *Cas9* gene. M: DL 5000 DNA marker; 1: negative control Longdao16; 2: positive control of transgenic L16-ehd1-#3 T_0 plant; 3-96: T_1 generation plants of L16-ehd1-#3.
