# Supplementary material for: In-Frame and Frame-Shift Editing of the Ehd1 Gene to Develop Japonica Rice With Prolonged Basic Vegetative Growth Periods
Source: Front Plant Sci. 2020 Mar 19;11:307. doi: 10.3389/fpls.2020.00307 (PMC7096585; doi:10.3389/fpls.2020.00307)
Supplement: Supplementary file 3 [file Data_Sheet_3.PDF]

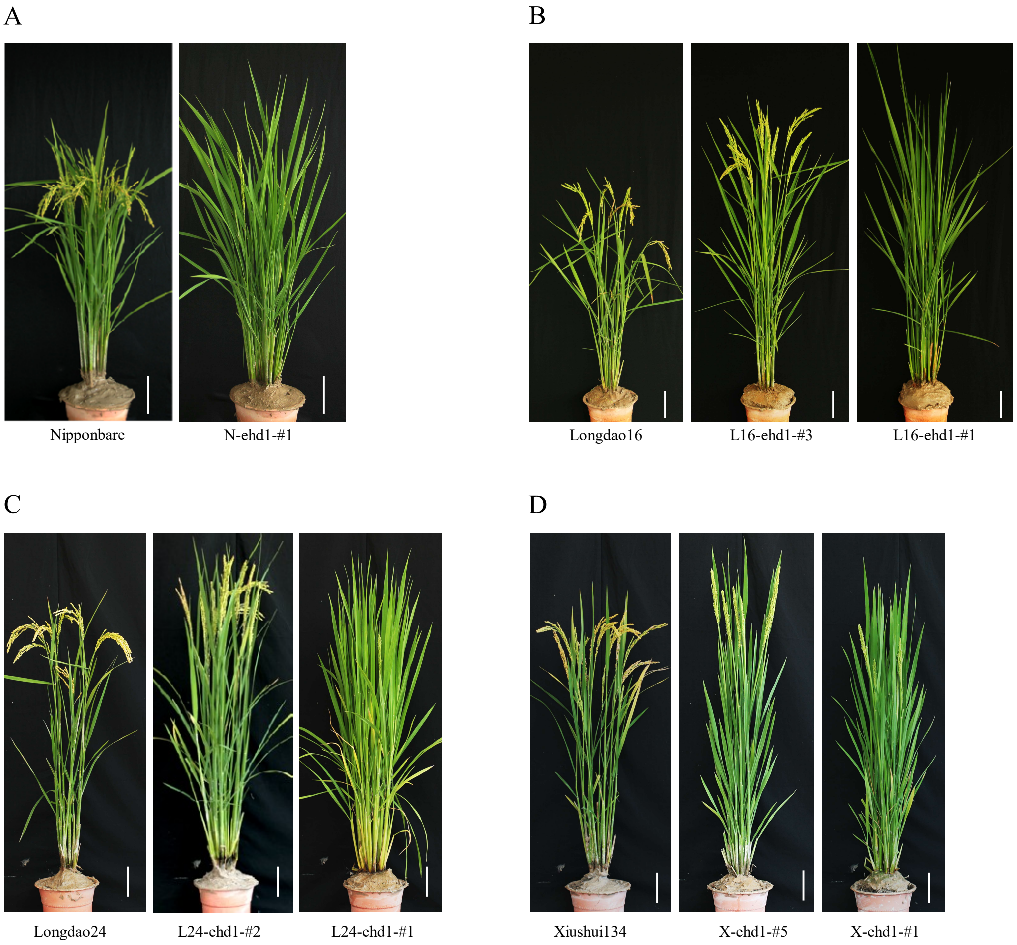

Supplementary Figure S3. *ehd1* mutant lines exhibited prolonged BVG periods. (A, B,C, D) Phenotypes of *ehd1* mutant lines in Nipponbare (A), Longdao16 (B), Longdao24 (C), and Xiushui134 (D) backgrounds, respectively. Pictures were taken at Fuzhou experimental station under long day condition. Scale bar: 10 cm.
