# Supplementary material for: In-Frame and Frame-Shift Editing of the Ehd1 Gene to Develop Japonica Rice With Prolonged Basic Vegetative Growth Periods
Source: Front Plant Sci. 2020 Mar 19;11:307. doi: 10.3389/fpls.2020.00307 (PMC7096585; doi:10.3389/fpls.2020.00307)
Supplement: Supplementary file 4 [file Data_Sheet_4.PDF]

A

```
Wide Type  : ATGGATCACCGAGAGCTGTGGCCTTATGGACTAAGAGTTCTGGTCATCGATGACGACTGTTCATACTTGTCAGTCATGGA  80
L16-ehd1-#3: ATGGATCACCGAGAGCTGTGGCCTTATG---------TTCTGGTCATCGATGACGACTGTTCATACTTGTCAGTCATGGA  71
L16-ehd1-#6: ATGGATCACCGAGAGCTGTGGCCTTATGG------TTTTCTGGTCATCGATGACGACTGTTCATACTTGTCAGTCATGGA  74
L24-ehd1-#2: ATGGATCACCGAGAGCTGTGGCCTTATGGAC------TTCTGGTCATCGATGACGACTGTTCATACTTGTCAGTCATGGA  74
L24-ehd1-#4: ATGGATCACCGAGAGCTGTGGCCTTATGGACTAAG---TCTGGTCATCGATGACGACTGTTCATACTTGTCAGTCATGGA  77
L24-ehd1-#8: ATGGATCACCGAGAGCTGTGGCCTTATGGAC---------TGGTCATCGATGACGACTGTTCATACTTGTCAGTCATGGA  71
X-ehd1-#5  : ATGGATCACCGAGAGCTGTGGCCTTATGGACTAA---TTCTGGTCATCGATGACGACTGTTCATACTTGTCAGTCATGGA  77

Wide Type  : AGATTTACTTCTGAAGTGCAGCTACAAGGTTACAACGTATAAGAACGTCAGAGAAGCTGTGCCTTTCATATTGGACAATC  160
L16-ehd1-#3: AGATTTACTTCTGAAGTGCAGCTACAAGGTTACAACGTATAAGAACGTCAGAGAAGCTGTGCCTTTCATATTGGACAATC  151
L16-ehd1-#6: AGATTTACTTCTGAAGTGCAGCTACAAGGTTACAACGTATAAGAACGTCAGAGAAGCTGTGCCTTTCATATTGGACAATC  154
L24-ehd1-#2: AGATTTACTTCTGAAGTGCAGCTACAAGGTTACAACGTATAAGAACGTCAGAGAAGCTGTGCCTTTCATATTGGACAATC  154
L24-ehd1-#4: AGATTTACTTCTGAAGTGCAGCTACAAGGTTACAACGTATAAGAACGTCAGAGAAGCTGTGCCTTTCATATTGGACAATC  157
L24-ehd1-#8: AGATTTACTTCTGAAGTGCAGCTACAAGGTTACAACGTATAAGAACGTCAGAGAAGCTGTGCCTTTCATATTGGACAATC  151
X-ehd1-#5  : AGATTTACTTCTGAAGTGCAGCTACAAGGTTACAACGTATAAGAACGTCAGAGAAGCTGTGCCTTTCATATTGGACAATC  157

Wide Type  : CACAAATAGTTGACCTAGTAATCAGTGATGCGTTCTTTCCTACCGAAGATGGTTTGCTCATTCTGCAAGAAGTAACCTCC  240
L16-ehd1-#3: CACAAATAGTTGACCTAGTAATCAGTGATGCGTTCTTTCCTACCGAAGATGGTTTGCTCATTCTGCAAGAAGTAACCTCC  231
L16-ehd1-#6: CACAAATAGTTGACCTAGTAATCAGTGATGCGTTCTTTCCTACCGAAGATGGTTTGCTCATTCTGCAAGAAGTAACCTCC  234
L24-ehd1-#2: CACAAATAGTTGACCTAGTAATCAGTGATGCGTTCTTTCCTACCGAAGATGGTTTGCTCATTCTGCAAGAAGTAACCTCC  234
L24-ehd1-#4: CACAAATAGTTGACCTAGTAATCAGTGATGCGTTCTTTCCTACCGAAGATGGTTTGCTCATTCTGCAAGAAGTAACCTCC  237
L24-ehd1-#8: CACAAATAGTTGACCTAGTAATCAGTGATGCGTTCTTTCCTACCGAAGATGGTTTGCTCATTCTGCAAGAAGTAACCTCC  231
X-ehd1-#5  : CACAAATAGTTGACCTAGTAATCAGTGATGCGTTCTTTCCTACCGAAGATGGTTTGCTCATTCTGCAAGAAGTAACCTCC  237

Wide Type  : CAAGTTGCCAGTCATCTGCAGAAATACAGGATGCAACTGAAGAAATCGATTCCAACAACAAGCAAACACGGAGCGACTTT  800
L16-ehd1-#3: CAAGTTGCCAGTCATCTGCAGAAATACAGGATGCAACTGAAGAAATCGATTCCAACAACAAGCAAACACGGAGCGACTTT  791
L16-ehd1-#6: CAAGTTGCCAGTCATCTGCAGAAATACAGGATGCAACTGAAGAAATCGATTCCAACAACAAGCAAACACGGAGCGACTTT  794
L24-ehd1-#2: CAAGTTGCCAGTCATCTGCAGAAATACAGGATGCAACTGAAGAAATCGATTCCAACAACAAGCAAACACGGAGCGACTTT  794
L24-ehd1-#4: CAAGTTGCCAGTCATCTGCAGAAATACAGGATGCAACTGAAGAAATCGATTCCAACAACAAGCAAACACGGAGCGACTTT  797
L24-ehd1-#8: CAAGTTGCCAGTCATCTGCAGAAATACAGGATGCAACTGAAGAAATCGATTCCAACAACAAGCAAACACGGAGCGACTTT  791
X-ehd1-#5  : CAAGTTGCCAGTCATCTGCAGAAATACAGGATGCAACTGAAGAAATCGATTCCAACAACAAGCAAACACGGAGCGACTTT  797

Wide Type  : GTCATCCACCGCTCTCGACAAAACACAAGACCACCCTTCAAGATCGCAGTATTTCAATCAAGACGGATGCAAGGAAATCA  880
L16-ehd1-#3: GTCATCCACCGCTCTCGACAAAACACAAGACCACCCTTCAAGATCGCAGTATTTCAATCAAGACGGATGCAAGGAAATCA  871
L16-ehd1-#6: GTCATCCACCGCTCTCGACAAAACACAAGACCACCCTTCAAGATCGCAGTATTTCAATCAAGACGGATGCAAGGAAATCA  874
L24-ehd1-#2: GTCATCCACCGCTCTCGACAAAACACAAGACCACCCTTCAAGATCGCAGTATTTCAATCAAGACGGATGCAAGGAAATCA  874
L24-ehd1-#4: GTCATCCACCGCTCTCGACAAAACACAAGACCACCCTTCAAGATCGCAGTATTTCAATCAAGACGGATGCAAGGAAATCA  877
L24-ehd1-#8: GTCATCCACCGCTCTCGACAAAACACAAGACCACCCTTCAAGATCGCAGTATTTCAATCAAGACGGATGCAAGGAAATCA  871
X-ehd1-#5  : GTCATCCACCGCTCTCGACAAAACACAAGACCACCCTTCAAGATCGCAGTATTTCAATCAAGACGGATGCAAGGAAATCA  877

Wide Type  : TGGACTACTCTTTACCGAGAGATGACCTCTCAAGTGGCTCAGAGTGCATGCTTGAAGAACTGAACGATTACTCATCCGAA  960
L16-ehd1-#3: TGGACTACTCTTTACCGAGAGATGACCTCTCAAGTGGCTCAGAGTGCATGCTTGAAGAACTGAACGATTACTCATCCGAA  951
L16-ehd1-#6: TGGACTACTCTTTACCGAGAGATGACCTCTCAAGTGGCTCAGAGTGCATGCTTGAAGAACTGAACGATTACTCATCCGAA  954
L24-ehd1-#2: TGGACTACTCTTTACCGAGAGATGACCTCTCAAGTGGCTCAGAGTGCATGCTTGAAGAACTGAACGATTACTCATCCGAA  954
L24-ehd1-#4: TGGACTACTCTTTACCGAGAGATGACCTCTCAAGTGGCTCAGAGTGCATGCTTGAAGAACTGAACGATTACTCATCCGAA  957
L24-ehd1-#8: TGGACTACTCTTTACCGAGAGATGACCTCTCAAGTGGCTCAGAGTGCATGCTTGAAGAACTGAACGATTACTCATCCGAA  951
X-ehd1-#5  : TGGACTACTCTTTACCGAGAGATGACCTCTCAAGTGGCTCAGAGTGCATGCTTGAAGAACTGAACGATTACTCATCCGAA  957

Wide Type  : GGTTTCCAAGATTTCCGATGGGATTCAGACAAACAGGAATATGGACCATGTTTTTGGAATTTCTAG  1026
L16-ehd1-#3: GGTTTCCAAGATTTCCGATGGGATTCAGACAAACAGGAATATGGACCATGTTTTTGGAATTTCTAG  1017
L16-ehd1-#6: GGTTTCCAAGATTTCCGATGGGATTCAGACAAACAGGAATATGGACCATGTTTTTGGAATTTCTAG  1020
L24-ehd1-#2: GGTTTCCAAGATTTCCGATGGGATTCAGACAAACAGGAATATGGACCATGTTTTTGGAATTTCTAG  1020
L24-ehd1-#4: GGTTTCCAAGATTTCCGATGGGATTCAGACAAACAGGAATATGGACCATGTTTTTGGAATTTCTAG  1023
L24-ehd1-#8: GGTTTCCAAGATTTCCGATGGGATTCAGACAAACAGGAATATGGACCATGTTTTTGGAATTTCTAG  1017
X-ehd1-#5  : GGTTTCCAAGATTTCCGATGGGATTCAGACAAACAGGAATATGGACCATGTTTTTGGAATTTCTAG  1023
```

Supplementary Figure S4. Multiple alignment of ORF nucleotide sequences (A) and amino acid sequences (B) of wild type *Ehd1* and the in-frame mutated alleles. Deletions and substitutions are indicated by dashes and blue letters, respectively.

B

```
                            D17                  Receiver domain              D63
Wide Type    : MDHRELWPYGLRVLVIDDDCSYLSVMEDLLLKCSYKVTTYKNVREAVPFILDNPQIVDLVISDAFFPTEDGLLILQEVTS : 80
L16-ehd1-#3 : MDHRELWPY---VLVIDDDCSYLSVMEDLLLKCSYKVTTYKNVREAVPFILDNPQIVDLVISDAFFPTEDGLLILQEVTS : 77
L16-ehd1-#6 : MDHRELWPYG--FLVIDDDCSYLSVMEDLLLKCSYKVTTYKNVREAVPFILDNPQIVDLVISDAFFPTEDGLLILQEVTS : 78
L24-ehd1-#2 : MDHRELWPYGL--LVIDDDCSYLSVMEDLLLKCSYKVTTYKNVREAVPFILDNPQIVDLVISDAFFPTEDGLLILQEVTS : 78
L24-ehd1-#4 : MDHRELWPYGL-SLVIDDDCSYLSVMEDLLLKCSYKVTTYKNVREAVPFILDNPQIVDLVISDAFFPTEDGLLILQEVTS : 79
L24-ehd1-#8 : MDHRELWPYG---LVIDDDCSYLSVMEDLLLKCSYKVTTYKNVREAVPFILDNPQIVDLVISDAFFPTEDGLLILQEVTS : 77
X-ehd1-#5    : MDHRELWPYGL-ILVIDDDCSYLSVMEDLLLKCSYKVTTYKNVREAVPFILDNPQIVDLVISDAFFPTEDGLLILQEVTS : 79

                                                   K117
Wide Type    : KFGIPTVIMASSGDTNTVMKYVANGAFDFLLKPVRIEELSNIWQHIFRKQMQDHKNNNMVGNLEKPGHPPSILAMARATP : 160
L16-ehd1-#3 : KFGIPTVIMASSGDTNTVMKYVANGAFDFLLKPVRIEELSNIWQHIFRKQMQDHKNNNMVGNLEKPGHPPSILAMARATP : 157
L16-ehd1-#6 : KFGIPTVIMASSGDTNTVMKYVANGAFDFLLKPVRIEELSNIWQHIFRKQMQDHKNNNMVGNLEKPGHPPSILAMARATP : 158
L24-ehd1-#2 : KFGIPTVIMASSGDTNTVMKYVANGAFDFLLKPVRIEELSNIWQHIFRKQMQDHKNNNMVGNLEKPGHPPSILAMARATP : 158
L24-ehd1-#4 : KFGIPTVIMASSGDTNTVMKYVANGAFDFLLKPVRIEELSNIWQHIFRKQMQDHKNNNMVGNLEKPGHPPSILAMARATP : 159
L24-ehd1-#8 : KFGIPTVIMASSGDTNTVMKYVANGAFDFLLKPVRIEELSNIWQHIFRKQMQDHKNNNMVGNLEKPGHPPSILAMARATP : 157
X-ehd1-#5    : KFGIPTVIMASSGDTNTVMKYVANGAFDFLLKPVRIEELSNIWQHIFRKQMQDHKNNNMVGNLEKPGHPPSILAMARATP : 159

                                                                         GARP
Wide Type    : ATTRSTATEASLAPLENEVRDDMVNYNGEITDIRDLGKSRLTWTTQLHRQFIAAVNHLGEDKAVPKKILGIMKVKHLTRE : 240
L16-ehd1-#3 : ATTRSTATEASLAPLENEVRDDMVNYNGEITDIRDLGKSRLTWTTQLHRQFIAAVNHLGEDKAVPKKILGIMKVKHLTRE : 237
L16-ehd1-#6 : ATTRSTATEASLAPLENEVRDDMVNYNGEITDIRDLGKSRLTWTTQLHRQFIAAVNHLGEDKAVPKKILGIMKVKHLTRE : 238
L24-ehd1-#2 : ATTRSTATEASLAPLENEVRDDMVNYNGEITDIRDLGKSRLTWTTQLHRQFIAAVNHLGEDKAVPKKILGIMKVKHLTRE : 238
L24-ehd1-#4 : ATTRSTATEASLAPLENEVRDDMVNYNGEITDIRDLGKSRLTWTTQLHRQFIAAVNHLGEDKAVPKKILGIMKVKHLTRE : 239
L24-ehd1-#8 : ATTRSTATEASLAPLENEVRDDMVNYNGEITDIRDLGKSRLTWTTQLHRQFIAAVNHLGEDKAVPKKILGIMKVKHLTRE : 237
X-ehd1-#5    : ATTRSTATEASLAPLENEVRDDMVNYNGEITDIRDLGKSRLTWTTQLHRQFIAAVNHLGEDKAVPKKILGIMKVKHLTRE : 239

Wide Type    : QVASHLQKYRMQLKKSIPTTSKHGATLSSTALDKTQDHPSRSQYFNQDGCKEIMDYSLPRDDLSSGSECMLEELNDYSSE : 320
L16-ehd1-#3 : QVASHLQKYRMQLKKSIPTTSKHGATLSSTALDKTQDHPSRSQYFNQDGCKEIMDYSLPRDDLSSGSECMLEELNDYSSE : 317
L16-ehd1-#6 : QVASHLQKYRMQLKKSIPTTSKHGATLSSTALDKTQDHPSRSQYFNQDGCKEIMDYSLPRDDLSSGSECMLEELNDYSSE : 318
L24-ehd1-#2 : QVASHLQKYRMQLKKSIPTTSKHGATLSSTALDKTQDHPSRSQYFNQDGCKEIMDYSLPRDDLSSGSECMLEELNDYSSE : 318
L24-ehd1-#4 : QVASHLQKYRMQLKKSIPTTSKHGATLSSTALDKTQDHPSRSQYFNQDGCKEIMDYSLPRDDLSSGSECMLEELNDYSSE : 319
L24-ehd1-#8 : QVASHLQKYRMQLKKSIPTTSKHGATLSSTALDKTQDHPSRSQYFNQDGCKEIMDYSLPRDDLSSGSECMLEELNDYSSE : 317
X-ehd1-#5    : QVASHLQKYRMQLKKSIPTTSKHGATLSSTALDKTQDHPSRSQYFNQDGCKEIMDYSLPRDDLSSGSECMLEELNDYSSE : 319

Wide Type    : GFQDFRWDSDKQEYGPCFWNF : 341
L16-ehd1-#3 : GFQDFRWDSDKQEYGPCFWNF : 338
L16-ehd1-#6 : GFQDFRWDSDKQEYGPCFWNF : 339
L24-ehd1-#2 : GFQDFRWDSDKQEYGPCFWNF : 339
L24-ehd1-#4 : GFQDFRWDSDKQEYGPCFWNF : 340
L24-ehd1-#8 : GFQDFRWDSDKQEYGPCFWNF : 338
X-ehd1-#5    : GFQDFRWDSDKQEYGPCFWNF : 340
```

Supplementary Figure S4. (continued). Multiple alignment of ORF nucleotide sequences (A) and amino acid sequences (B) of wild type *Ehd1* and the in-frame mutated alleles. Deletions and substitutions are indicated by dashes and blue letters, respectively. The receiver domain is highlighted in red fonts, and the GARP domain is highlighted in green fonts on a yellow background. The conserved D-D-K (D17, D63 and K117) motifs needed for phosphorylation of receiver domain are in black boxes.
