# Supplementary material for: In-Frame and Frame-Shift Editing of the Ehd1 Gene to Develop Japonica Rice With Prolonged Basic Vegetative Growth Periods
Source: Front Plant Sci. 2020 Mar 19;11:307. doi: 10.3389/fpls.2020.00307 (PMC7096585; doi:10.3389/fpls.2020.00307)
Supplement: Supplementary file 5 [file Data_Sheet_5.PDF]

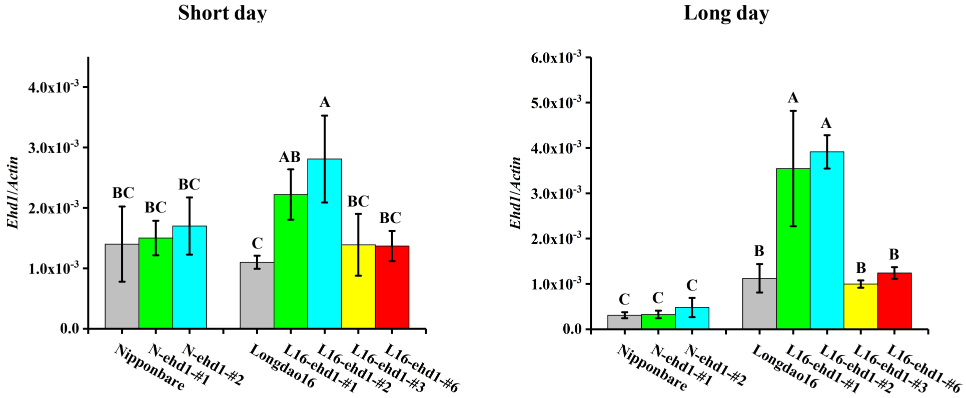

Supplementary Figure S5. mRNA expression levels of *Ehd1* in the frame-shift mutant lines N-ehd1-#1, N-ehd1-#2, L16-end1-#1, and L16-end1-#2, and in-frame mutant lines L16-end1-#3 and L16-end1-#6 at 50 d after sowing. T_2 homozygous lines were used for detection. Fully expanded leaves from three different rice plants were sampled 2 h after dawn, three independent plants were used as biological replicates. mRNA expression levels are shown as mean and standard deviation. The letters A, B, and C indicate significant differences according to LSD multiple range test at $P \leq 0.01$.
