# Supplementary material for: In-Frame and Frame-Shift Editing of the Ehd1 Gene to Develop Japonica Rice With Prolonged Basic Vegetative Growth Periods
Source: Front Plant Sci. 2020 Mar 19;11:307. doi: 10.3389/fpls.2020.00307 (PMC7096585; doi:10.3389/fpls.2020.00307)
Supplement: Supplementary file 7 [file Data_Sheet_7.PDF]

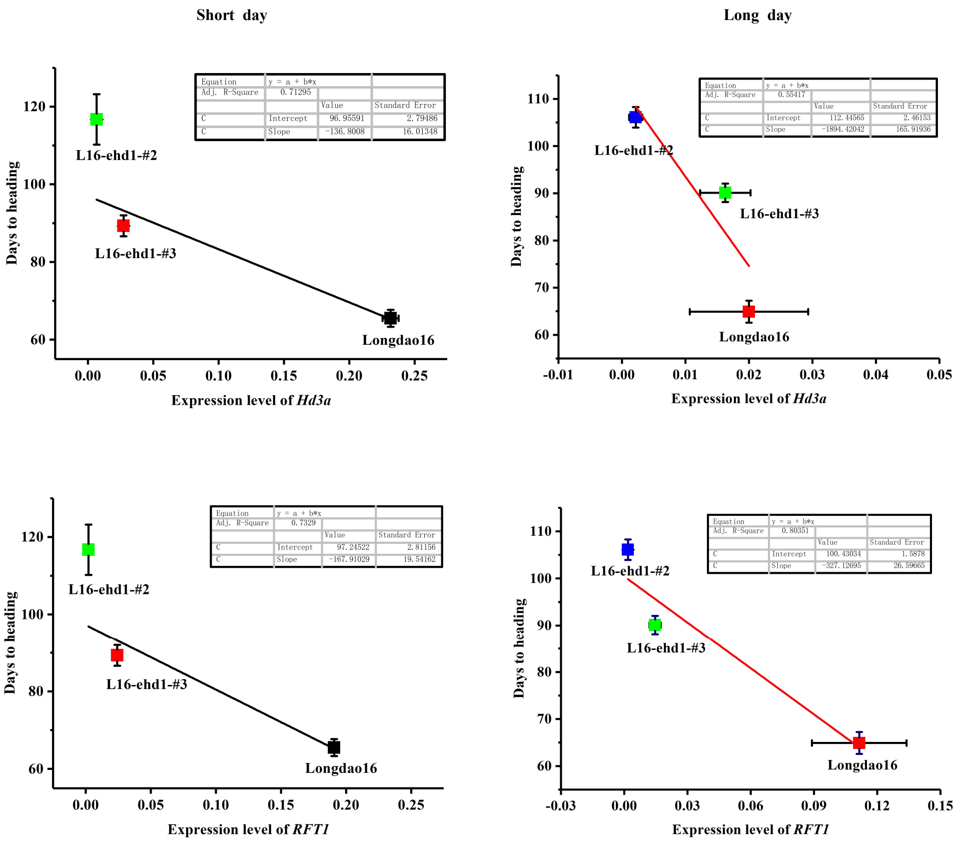

Supplementary Figure S7. Liner fitting analysis of the relationship between heading dates and expression levels of *Hd3a* and *RFT1* in L16-ehd1-#2, L16-ehd1-#3 and Longdao16 under both SD and LD conditions. The first fully expanded leaves of 40 days seedlings were collected for qRT-PCR analysis.
