# Supplementary material for: In-Frame and Frame-Shift Editing of the Ehd1 Gene to Develop Japonica Rice With Prolonged Basic Vegetative Growth Periods
Source: Front Plant Sci. 2020 Mar 19;11:307. doi: 10.3389/fpls.2020.00307 (PMC7096585; doi:10.3389/fpls.2020.00307)
Supplement: Supplementary file 8 [file Data_Sheet_8.PDF]

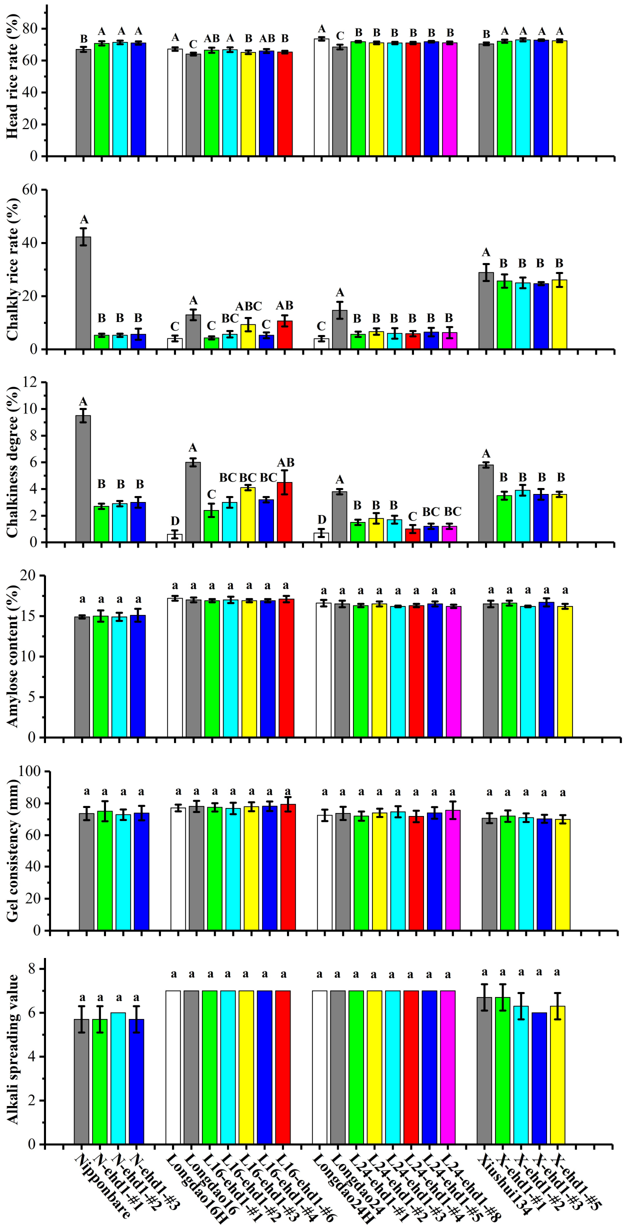

Supplementary Figure S8. Evaluation of grain quality in the *ehd1* mutant lines. Grains from Nipponbare, Longdao16, Longdao24, Xiushui134 and the *ehd1* mutant lines cultivated in Fuzhou, Fujian Province, and from the two Heilongjiang varieties Longdao16 and Longdao24 (referred to as Longdao16H and Longdao24H, respectively) cultivated in Haerbing, Heilongjiang Province (from April to September) were evaluated. The rice lines cultivated in Fuzhou were sowed on 18 May. Nipponbare and Longdao16 started to flower around 21 July; Longdao24 and Xiushui134 started to flower around 29 July and around 10 August, respectively. The three frame-shift lines of Nipponbare (N-ehd1-#1/-#2/-#3), and the six in-frame deletion lines of Longdao16 (L16-ehd1-#3/-#6), Longdao24 (L24-ehd1-#2/-#4/-#8), and Xiushui134 (X-ehd1-#5) started to flower around 20 August, whereas the frame-shift lines of Longdao16 (L16-ehd1-#1/-#2/-#4), Longdao24 (L24-ehd1-#1/-#3/-#5), and Xiushui134 (X-ehd1-#1/-#2/-#3) started to flower around 1 to 5 September. The average temperatures during grain-filling stage of Nipponbare, Longdao16, Longdao24, and Xiushui134 were around 27.5°C to 26.4°C; whereas the average temperatures were about 25.5°C to 25.0°C during grain-filling stages of N-ehd1-#1/-#2/-#3, L16-ehd1-#3/-#6, L24-ehd1-#2/-#4/-#8, X-ehd1-#5, and of L16-ehd1-#1/-#2/-#4, L24-ehd1-#1/-#3/-#5, X-ehd1-#1/-#2/-#3. While high temperature during grain-filling stage led to higher chalk values of Nipponbare, Longdao16, Longdao24, or Xiushui134, the delay of flowering time allowed the *ehd1* mutant lines to be exposed to relatively lower temperatures during grain-filling stage, resulting in higher head rice rate, lower chalky rice rate and chalkiness degree compared with wild-types.
