# Supplementary material for: In-Frame and Frame-Shift Editing of the Ehd1 Gene to Develop Japonica Rice With Prolonged Basic Vegetative Growth Periods
Source: Front Plant Sci. 2020 Mar 19;11:307. doi: 10.3389/fpls.2020.00307 (PMC7096585; doi:10.3389/fpls.2020.00307)
Supplement: Supplementary file 9 [file Table_1.docx]

**Supplementary Table S1.** Primers used in this study

| **Primer** | **Sequence (5′-3′)** | **Comment** |
| --- | --- | --- |
| ED1-F | CAGGCCTTATGGACTAAGAGTTC | F primer for making sgRNA-Cas9 construct *ED1* |
| ED1-R | AACGAACTCTTAGTCCATAAGGC | R primer for making sgRNA-Cas9 construct *ED1* |
| Ehd1-F | CGGAGAAGCAGATGTAGTCG | F primer for genotyping *Ehd1* target mutation sequence |
| Ehd1-R | GACAAGTATGAACAGTCGTC | R primer for genotyping *Ehd1* target mutation sequence |
| Ehd1-RT-F | GATCATATCAACGGTGTTATCC | F primer for amplifying cDNA sequence of *Ehd1/rd*-*Ehd1* |
| Ehd1-RT-R | GATGTGGTAATGTTCACTTATTCA | R primer for amplifying cDNA sequence of *Ehd1*/*rd*-*Ehd1* |
| Ehd1-RT-F2 | GCGCTTCTGATTTCCTGC | F primer for real-time qPCR of *Ehd1* |
| Ehd1-RT-R2 | CGGAATATGTGCTGCCAG | R primer for real-time qPCR of *Ehd1* |
| Actin-F | CGTCTGCGATAATGGAACTG | F primer for real-time qPCR of *Actin* |
| Actin-R | TCTGGGTCATCTTCTCACGA | R primer for real-time qPCR of *Actin* |
| Hd3a -F | GCTCACTATCATCATCCAGCATG | F primer for real-time qPCR of *Hd3a* |
| Hd3a- R | CCTTGCTCAGCTATTTAATTGCATAA | R primer for real-time qPCR of *Hd3a* |
| RFT1-F | CCGTCTACTTCAACTGCCAG | F primer for real-time qPCR of *RFT1* |
| RFT1-R | GTCTCAGCTTAGCTATAGCT | R primer for real-time qPCR of *RFT1* |
| CZT-F | GGGAGATCCAGCTAGAGGTC | F primer for detecting the *Cas9* gene |
| CZT-R | GGAAGGAGGAAGACAAGG | R primer for detecting the *Cas9* gene |
| Hpt-F | AGGTCAGGCTCTCGCTAAAC | F primer for detecting the *hygromycin phosphotransferase* gene (*Hpt*) |
| Hpt-R | ACGTAAGGGATGACGCACAAT | R primer for detecting the *hygromycin phosphotransferase* gene (*Hpt*) |
| OTF1 | TTGTACTTGTTCATCCCGTCG | F primer for detecting the putative off-target site of S1 |
| OTR1 | AGCAGCCTTATACTCCCTCAG | R primer for detecting the putative off-target site of S1 |
| OTF2 | GGAGGGGTTGTACTTGTTCA | F primer for detecting the putative off-target site of S2 |
| OTR2 | TGGCACTGCGATGGAGC | R primer for detecting the putative off-target site of S2 |
| OTF3 | TTGTACTTGTTCATCCCGTC | F primer for detecting the putative off-target site of S3 |
| OTR3 | GAGAACCAACTTCCAAAATAAAA | R primer for detecting the putative off-target site of S3 |
| OTF4 | TCAAACAGATTCAGTCGTGG | F primer for detecting the putative off-target site of S4 |
| OTR4 | TGCTATCTCATTCGGTCACTC | R primer for detecting the putative off-target site of S4 |
| OTF5 | TCCATAGTCCTTTGGCTTGA | F primer for detecting the putative off-target site of S5 |
| OTR5 | GGTTGCTGCGATGTTTCT | R primer for detecting the putative off-target site of S5 |
| OTF6 | AAGTTACCTCCTTGATAGTTCTG | F primer for detecting the putative off-target site of S6 |
| OTR6 | GGCAAATGGTTAATCCTCG | R primer for detecting the putative off-target site of S6 |
