# Supplementary material for: In-Frame and Frame-Shift Editing of the Ehd1 Gene to Develop Japonica Rice With Prolonged Basic Vegetative Growth Periods
Source: Front Plant Sci. 2020 Mar 19;11:307. doi: 10.3389/fpls.2020.00307 (PMC7096585; doi:10.3389/fpls.2020.00307)
Supplement: Supplementary file 10 [file Table_2.DOCX]

**Supplementary Table S2.** Mutations in 53 T_0_ independent transgenic plants

| Background | Line | Mutation sequences* | Mutation types† | Zygosity |
| --- | --- | --- | --- | --- |
| Nipponbare | N-ehd1-#1 | ccttatggactaaga-ttctgg | -1 | Homozygous |
|  | N-ehd1-#2 | ccttatggactaag--ttctgg | -2/wt | Heterozygous |
|  | N-ehd1-#3 | ccttatggactaagagTttctgg | +1 | Homozygous |
|  | N-ehd1-#4 | ccttatggactaagag(A/T)ttctgg | +1/+1 | Bi-allelic |
|  | N-ehd1-#5 | ccttatggactaagagTttctgg | +1/wt | Heterozygous |
|  | N-ehd1-#6 | ccttatggactaagagAttctgg | +1/wt | Heterozygous |
|  | N-ehd1-#7 | ccttatggactaagagGttctgg | +1 | Homozygous |
|  | N-ehd1-#8 | ccttatggactaagag(G/C)ttctgg | +1/+1 | Bi-allelic |
|  | N-ehd1-#9 | ccttatggactaaga-ttctgg | -1/wt | Heterozygous |
|  | N-ehd1-#10 | ccttatggactaaga(-/gT)ttctgg | -1/+1 | Bi-allelic |
|  | N-ehd1-#11 | ccttatggactaagagTttctgg | +1/wt | Heterozygous |
|  | N-ehd1-#12 | ccttatggactaagagCttctgg | +1/wt | Heterozygous |
|  | N-ehd1-#13 | ccttatggactaagag(A/T)ttctgg | +1/+1 | Bi-allelic |
|  | N-ehd1-#14 | ccttatggactaagagAttctgg | +1/wt | Heterozygous |
| Longdao16 | L16-ehd1-#1 | ccttatggactaaga-ttctgg | -1/wt | Heterozygous |
|  | L16-ehd1-#2 | ccttatggactaagagAttctgg | +1 | Homozygous |
|  | L16-ehd1-#3 | ccttatg---------ttctgg | -9/wt | Heterozygous |
|  | L16-ehd1-#4 | ccttatggactaagagCttctgg | +1/wt | Heterozygous |
|  | L16-ehd1-#5 | ccttatggactaagag(A/T)ttctgg | +1/+1 | Bi-allelic |
|  | L16-ehd1-#6 | ccttatgg--------TTttctgg | -8&+2 | Homozygous |
|  | L16-ehd1-#7 | ccttatggactaagag(G/C)ttctgg | +1/+1 | Bi-allelic |
|  | L16-ehd1-#8 | ccttatggactaagagGttctgg | +1/wt | Heterozygous |
|  | L16-ehd1-#9 | ccttatggactaagagAttctgg | +1 | Homozygous |
|  | L16-ehd1-#10 | ccttatggactaagagTttctgg | +1/wt | Heterozygous |
|  | L16-ehd1-#11 | ccttatggactaagag(A/T)ttctgg | +1/+1 | Bi-allelic |
|  | L16-ehd1-#12 | ccttatggactaaga-ttctgg | -1/wt | Heterozygous |
|  | L16-ehd1-#13 | ccttatggactaagagAttctgg | +1/wt | Heterozygous |
|  | L16-ehd1-#14 | ccttatggactaagagCttctgg | +1 | Homozygous |
|  | L16-ehd1-#15 | ccttatggactaagagAttctgg | +1/wt | Heterozygous |
| Longdao24 | L24-ehd1-#1 | ccttatggactaagagCttctgg | +1 | Homozygous |
|  | L24-ehd1-#2 | ccttatggac------ttctgg | -6/wt | Heterozygous |
|  | L24-ehd1-#3 | ccttatggactaagag(G/C)ttctgg | +1/+1 | Bi-allelic |
|  | L24-ehd1-#4 | ccttatggactaag---tctgg | -3/wt | Heterozygous |
|  | L24-ehd1-#5 | ccttatggactaagagAttctgg | +1/wt | Heterozygous |
|  | L24-ehd1-#6 | ccttatggactaagagTttctgg | +1 | Homozygous |
|  | L24-ehd1-#7 | ccttatggactaagag(-/C)ttctgg | -1/+1 | Bi-allelic |
|  | L24-ehd1-#8 | ccttatggact---------ggtcatc | -9/wt | Heterozygous |
|  | L24-ehd1-#9 | ccttatggactaaga-ttctgg | -1/wt | Heterozygous |
|  | L24-ehd1-#10 | ccttatggactaagagAttctgg | +1/wt | Heterozygous |
|  | L24-ehd1-#11 | ccttatggactaagag(A/T)ttctgg | +1/+1 | Bi-allelic |
|  | L24-ehd1-#12 | ccttatggactaagagTttctgg | +1/wt | Heterozygous |
|  | L24-ehd1-#13 | ccttatggactaagagCttctgg | +1/wt | Heterozygous |
| Xiushui134 | X-ehd1-#1 | ccttatggactaagagAttctgg | +1/wt | Heterozygous |
|  | X-ehd1-#2 | cct-------------ttctgg | -13/wt | Heterozygous |
|  | X-ehd1-#3 | ccttatggactaagagTttctgg | +1 | Homozygous |
|  | X-ehd1-#4 | ccttatggactaagagAttctgg | +1/wt | Heterozygous |
|  | X-ehd1-#5 | ccttatggactaa---ttctgg | -3/wt | Heterozygous |
|  | X-ehd1-#6 | ccttatggactaagagAttctgg | +1/wt | Heterozygous |
|  | X-ehd1-#7 | ccttatggactaagagGttctgg | +1 | Homozygous |
|  | X-ehd1-#8 | ccttatggactaagagTttctgg | +1/wt | Heterozygous |
|  | X-ehd1-#9 | ccttatggactaagag(-/C)ttctgg | -1/+1 | Bi-allelic |
|  | X-ehd1-#10 | ccttatggactaagagCttctgg | +1/wt | Heterozygous |
|  | X-ehd1-#11 | ccttatggactaagagCttctgg | +1/wt | Heterozygous |

*Deletions and insertions are indicated by dashes and red letters, respectively; Bi-allelic mutations are distinguished by ‘/’.

†+: insertion, -: deletion, and wt: wild type. The numbers indicate the number of nucleotides involved.
