# Supplementary material for: In-Frame and Frame-Shift Editing of the Ehd1 Gene to Develop Japonica Rice With Prolonged Basic Vegetative Growth Periods
Source: Front Plant Sci. 2020 Mar 19;11:307. doi: 10.3389/fpls.2020.00307 (PMC7096585; doi:10.3389/fpls.2020.00307)
Supplement: Supplementary file 11 [file Table_3.DOCX]

| Potential off-target site | Sequence of the potential off-target site* | Potential off-target locus | Gene | Region | No. of mismatching bases | No. of plants sequenced† | No. of plants with mutations |
| --- | --- | --- | --- | --- | --- | --- | --- |
| *ED1* | GCCTTATGGACTAAGAGTTCTGG |  |  |  |  |  |  |
| *OT1* | GCCTTAGGGACTTAGGGTTTGGG | Chr4: 14807992-14808014 | Os04g0321600 | UTR | 4 | 40 | 0 |
| *OT2* | GCCTTAGGGACTTAGGGTTTGGG | Chr8: 19307994-19308016 | Os08g0404300 | UTR | 4 | 40 | 0 |
| *OT3* | GCCTTAGGGACTTAGGGTTTGGG | Chr8: 19313149-19313171 |  | Intergenic | 4 | 40 | 0 |
| *OT4* | GCATCATGGACTAATAGTACTAG | Chr8: 27408256-27408278 |  | Intergenic | 5 | 40 | 0 |
| *OT5* | ACATTATGGACCAAGTGTTCAGG | Chr4: 787248-787270 | Os04g0112766 | UTR | 4 | 40 | 0 |
| *OT6* | ACATTATGGACCAAGTGTTCAGG | Chr11: 21097529-21097551 |  | Intergenic | 4 | 40 | 0 |

**Supplementary Table S3.** Evaluation for potential off-target mutations in T_2_ mutant lines

*The mismatching bases were shown in red color, and PAM sequences are underlined.

†Forty transgene-free T_2_ mutant plants in Nipponbare, Longdao16, Longdao24 or Xiushui134 backgrounds were evaluated (3 of N-ehd1-#1, 3 of N-ehd1-#2, 3 of N-ehd1-#3, 3 of L16-ehd1-#1, 2 of L16-ehd1-#2, 2 of L16-ehd1-#3, 2 of L16-ehd1-#4, 2 of L16-ehd1-#6, 2 of L24-ehd1-#1, 2 of L24-ehd1-#2, 2 of L24-ehd1-#3, 2 of L24-ehd1-#4, 2 of L24-ehd1-#8, 2 of X-ehd1-#1, 2 of X-ehd1-#2, 2 of X-ehd1-#3, 2 of X-ehd1-#4 and 2 of X-ehd1-#5. ).
